# Whole-body cellular mapping in mouse using standard IgG antibodies

**DOI:** 10.1038/s41587-023-01846-0

**Published:** 2023-07-10

**Authors:** Hongcheng Mai, Jie Luo, Luciano Hoeher, Rami Al-Maskari, Izabela Horvath, Ying Chen, Florian Kofler, Marie Piraud, Johannes C. Paetzold, Jennifer Modamio, Mihail Todorov, Markus Elsner, Farida Hellal, Ali Ertürk

**Affiliations:** 1 Institute for Tissue Engineering and Regenerative Medicine, Helmholtz Center Munich, Neuherberg, Germany; 2 Institute for Stroke and Dementia Research, Medical Centre of the University of Munich, Ludwig-Maximilians University of Munich, Munich, Germany; 3 Munich Medical Research School, Munich, Germany; 4 Deep Piction GmbH, Munich, Germany; 5 TUM School of Computation, Information and Technology, Technical University of Munich, Munich, Germany; 6 Faculty of Medicine, Ludwig-Maximilians University of Munich, Munich, Germany; 7 Helmholtz Al, Helmholtz Center Munich, Neuherberg, Germany; 8 Department of Informatics, Technical University of Munich, Munich, Germany; 9 TranslaTUM – Central Institute for Translational Cancer Research, Technical University of Munich, Munich, Germany; 10 Department of Diagnostic and Interventional Neuroradiology, School of Medicine, Klinikum rechts der Isar, Technical University of Munich, Munich, Germany; 11 Department of Computing, Imperial College London, London, UK; 12 Munich Cluster for Systems Neurology (SyNergy), Munich, Germany; 13 Graduate School of Neuroscience (GSN), Munich, Germany

**Keywords:** Optical imaging, Neuroscience

## Abstract

Whole-body imaging techniques play a vital role in exploring the interplay of physiological systems in maintaining health and driving disease. We introduce wildDISCO, a new approach for whole-body immunolabeling, optical clearing and imaging in mice, circumventing the need for transgenic reporter animals or nanobody labeling and so overcoming existing technical limitations. We identified heptakis(2,6-di-*O*-methyl)-β-cyclodextrin as a potent enhancer of cholesterol extraction and membrane permeabilization, enabling deep, homogeneous penetration of standard antibodies without aggregation. WildDISCO facilitates imaging of peripheral nervous systems, lymphatic vessels and immune cells in whole mice at cellular resolution by labeling diverse endogenous proteins. Additionally, we examined rare proliferating cells and the effects of biological perturbations, as demonstrated in germ-free mice. We applied wildDISCO to map tertiary lymphoid structures in the context of breast cancer, considering both primary tumor and metastases throughout the mouse body. An atlas of high-resolution images showcasing mouse nervous, lymphatic and vascular systems is accessible at http://discotechnologies.org/wildDISCO/atlas/index.php.

## Abstract (web summary)

Whole mice are immunolabeled with standard antibodies using wildDISCO.

## Main

More than a century of dedicated work has provided a detailed understanding of the gross anatomy of the human body and the body of common model organisms and has produced detailed histological maps of many individual organs. However, it remains challenging to map the distribution, connectivity and molecular makeup of cell types across the whole body for a given experimental condition. For instance, although the nervous system pervades every region of the mammalian body, cellular-level maps depicting the intricate web of nerves linking various organs and connecting them to the central nervous system are still lacking^1–3^. In addition, most methods to image nerves or other cells in the context of whole bodies rely on transgenic animals^4,5^, which limits the flexibility of experimental design. Generating transgenic animals to map changes in the distribution of relevant proteins is usually prohibitively expensive and time consuming. However, such whole-body connectivity maps will be needed to understand the functional interdependence of organ systems and how a disease starting in one part of the body affects distal organs and tissues, for example, during neurodegeneration or systemic inflammation.

Recent clearing methods have enabled labeling and imaging of intact tissues^6^, mouse organs^7^ and bodies^3,8–15^, parts of human organs^16^ and even human embryos^17^. While previous methods, such as CUBIC, PACT, PEGASOS and uDISCO, enabled whole-body imaging, they relied on transgenic expression of fluorescent proteins in a subset of cells, such as mice expressing Thy-1 enhanced green fluorescent protein (EGFP) in neurons^18^. For example, vDISCO^5^ relies on the small antibody fragments called nanobodies (roughly one-tenth of IgG size) for whole mouse body labeling. Despite the thousands of conventional antibodies that have been developed over the past decades, only a handful of nanobodies are available or function effectively in a histological context. Consequently, we still lack appropriate, universally applicable labeling methods for whole mouse bodies that use standard IgG antibodies.

Although homogeneous labeling of whole bodies with small molecules (for example, DNA-labeling dyes) or nanobodies can be achieved by cardiac pumping of solutions through the mouse vasculature^5^, standard IgG antibodies face several challenges. These include degradation or precipitation during perfusion, inability to consistently penetrate various tissue layers—including muscles and bones—and insufficient membrane permeabilization to reach deep into all tissues with diverse properties.

To overcome these challenges, we developed wildDISCO (immunolabeling of wildtype mice and DISCO clearing), a chemical method enhancing the penetration of standard (roughly 150 kDa in size) antibodies into the whole roughly 2 cm-thick mouse body. Our method relies on cholesterol extraction for permeabilization to ensure homogeneous penetration and staining across the tissues of the whole mouse body including muscles, bones, the brain and the spinal cord. Combining whole-body antibody labeling with DISCO-based tissue clearing allowed us to provide body-wide maps of cell-type and protein distribution with unprecedented ease and will help advance our understanding of biological systems.

## Results

### Development of wildDISCO

**Fig. 1:**
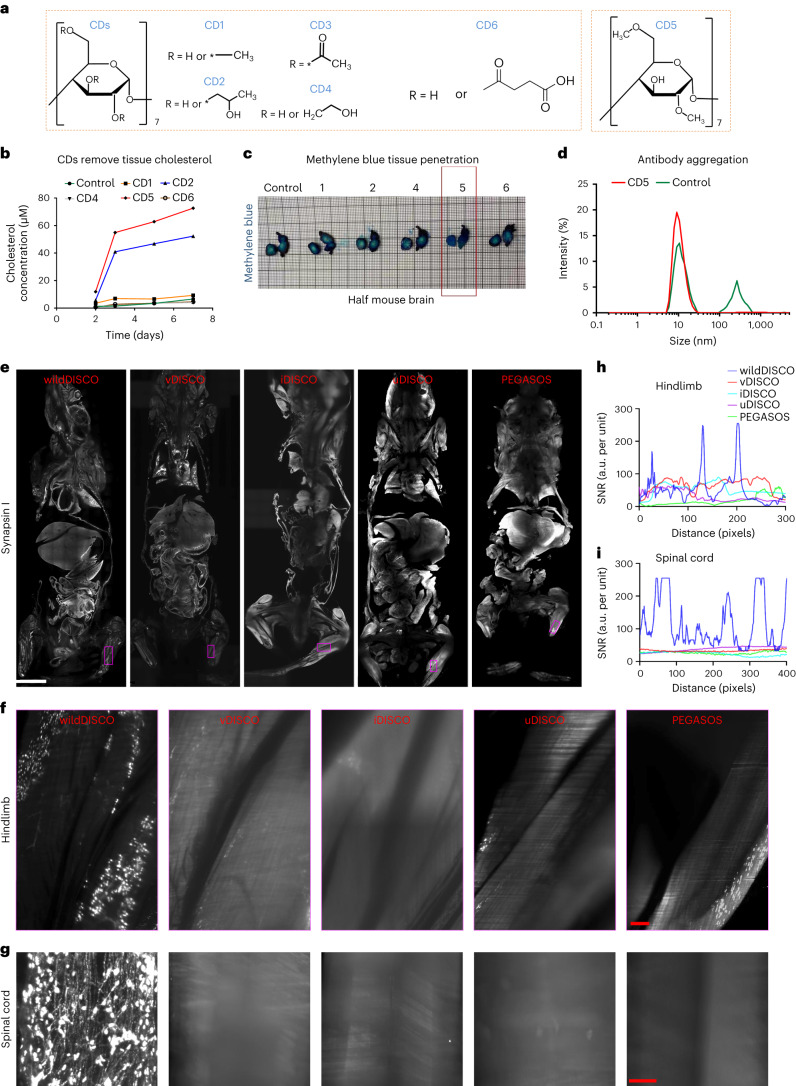
Exploring cyclodextrins as whole-body conventional IgG antibody staining chemicals and comparison of different clearing methods for whole mouse antibody staining. **a**, The structure of cyclodextrin (CD) with different substituent groups: CD1 (methyl-β-cyclodextrin), CD2 (2-hydroxypropyl-β-cyclodextrin), CD3 (triacetyl-β-cyclodextrin), CD4 ((2-hydroxyethyl)-β-cyclodextrin), CD5 (heptakis(2,6-di-*O*-methyl)-β-cyclodextrin) and CD6 (succinyl-β-cyclodextrin). **b**, Measurements of supernatant cholesterol concentration after different CD-containing buffer incubation on the seventh day for 25 mg mouse liver sections. **c**, Methylene blue staining of a single hemisphere of mouse brains after permeabilization with different CD-containing solutions. CD5 is shown to greatly enhance tissue permeabilization compared to others. **d**, DLS for size distribution of TH antibody in solutions with and without CD5. **e**–**i**, Comparison of different clearing methods for whole mouse body antibody staining. **e**, Optical 2D light-sheet microscopy images of the whole mouse body stained with synapsin 1 antibody by wildDISCO, vDISCO, iDISCO, uDISCO and PEGASOS methods, respectively. Scale bar, 5,000 μm. **f**,**g**, Representative 2D optical images of mouse hindlimb (**f**) and spinal cord (**g**) by wildDISCO, vDISCO, iDISCO, uDISCO and PEGASOS methods. Scale bars: **f**, 200 μm; **g**, 300 μm. *n* = 3. **h**,**i**, Quantification of antibody penetration depth into the hindlimb (**h**) and spinal cord (**i**) of mice by wildDISCO, vDISCO, iDISCO, uDISCO and PEGASOS methods. Source data

We hypothesized that poor cholesterol extraction from cell membranes might have been a factor limiting tissue permeabilization in previous methods. Cyclodextrin is a small oligosaccharide ring used to deplete cholesterol in live membranes^12^. We screened for β-cyclodextrin variants with a diverse nature and number of R-motifs (for example, methyl-, hydroxypropyl-, hydroxyethyl-, succinyl- and acetyl-) (Fig. 1a) for their potential ability to facilitate cholesterol extraction in fixed samples in combination with the CHAPS and Triton X-100 detergents to enhance permeabilization. Assessing cholesterol extraction using the cholesterol and cholesterol ester-glo assay, we found that the heptakis(2,6-di-*O*-methyl)-β-cyclodextrin (CD5) extracted most cholesterol from mouse liver tissue after 7 days (Fig. 1b). Addition of CD5 to the permeabilization reactions allowed rapid and homogeneous penetration of methylene blue into the whole mouse brain within 12 h, whereas other tested cyclodextrin chemicals allowed only limited penetration (Fig. 1c and Supplementary Fig. 1).

As cyclodextrins have been previously reported to stabilize proteins in solution by preventing aggregation^19^, we also measured the antibody size in antibody solutions using dynamic light scattering (DLS). We found that after 7 days at room temperature in a HEPES buffer without CD5, antibodies showed two peaks in the DLS data: one peak at 11.5 nm presumably for the antibody monomer and a peak for larger sizes, which most likely corresponds to different aggregation states. Thus, addition of CD5 prevented the formation of aggregates (Fig. 1d).

We next tested whether the enhanced membrane permeabilization and the decreased aggregation^20^ propensity of antibodies in the CD5-containing buffers translated into an increased homogeneity and depth of antibody staining in whole mouse bodies. We were able to demonstrate whole mouse labeling using active perfusion of the CD5-containing staining solution and called this protocol wildDISCO (Supplementary Fig. 2). We compared different cyclodextrins for kidney staining and found that CD5 supported the most uniform antibody penetration (Supplementary Fig. 3). Furthermore, we tested the penetration of an antibody against tyrosine hydroxylase (TH)^+^, a marker of sympathetic nerves, into the whole mouse body with and without CD5 using passive staining and found that antibodies quickly aggregated without CD5 (Supplementary Fig. 4). In contrast to active perfusion, passive staining yielded only partial antibody penetration. Using passive diffusion, we found few TH^+^ sympathetic nerves in the forelimb and no sympathetic nerve staining in the liver, indicating that antibody penetration requires perfusion through the mouse vasculature to achieve a homogeneous staining throughout the body. To determine the optimal perfusion time, we stained mice with a synapsin 1 antibody (Supplementary Fig. 5) for 1 and 7 days. While peripheral tissues such as the hindlimbs were already well stained after 1 day (Supplementary Fig. 5b,c,g,h), uniform staining of synapsin 1 in internal organs, such as the liver (Supplementary Fig. 5d,e,i,j), required a 7-day perfusion. Therefore, a 7-day active perfusion-based wildDISCO yielded the best results for most applications ensuring a complete antibody penetration and binding to the target antigens.

We evaluated wildDISCO against other established methods (vDISCO, iDISCO, uDISCO and PEGASOS) in terms of their efficacy in staining an entire mouse using standard antibodies. In a direct side-by-side comparison using the synapsin 1 antibody, we observed incomplete staining in addition to many blurry imaged organs (such as the head and hindlimbs) from iDISCO, uDISCO and PEGASOS stained samples (probably due to the lack of a decalcification step in these protocols) (Fig. 1e–g). Although vDISCO is able to stain whole mouse bodies with nanobodies, it has already been reported not to provide deep tissue staining with standard IgG antibodies^5^, as we confirmed in our new set of experiments. By contrast, wildDISCO enabled full penetration of antibodies homogeneously and deeply into whole mouse internal organs, such as hindlimbs, spinal cord, forelimbs, kidneys and liver (Fig. 1f–i and Supplementary Fig. 6).

To test its reproducibility, we applied wildDISCO at the same time on five different mice and labeled them with TH antibodies. Quantifying the sympathetic nerve intensity, we demonstrated that there was no statistical difference in the labeling between different mice, validating the high reproducibility of our wildDISCO methodology (Supplementary Fig. 7).

At present, reporter mice are predominantly used for imaging biological systems in entire mouse bodies, such as with whole-body clearing methods such as vDISCO. To investigate the accuracy of transgenic reporters in reflecting the biodistributions of the endogenous protein, we conducted a comparison with wildDISCO labeling. By labeling LYVE-1 EGFP reporter mice with LYVE-1 antibody using wildDISCO, we were able to show that the LYVE-1 transgenic reporter (which expresses EGFP under the LYVE-1 promoter from a construct integrated at a different genomic locus) only partially labels structures expressing the endogenous proteins (Supplementary Fig. 8). This suggests that transgenic reporters may not accurately represent the full distribution of endogenous proteins, underscoring the power of wildDISCO to ensure a more faithful portrayal of protein distribution in biological systems.

### wildDISCO labels body-wide systems

**Fig. 2:**
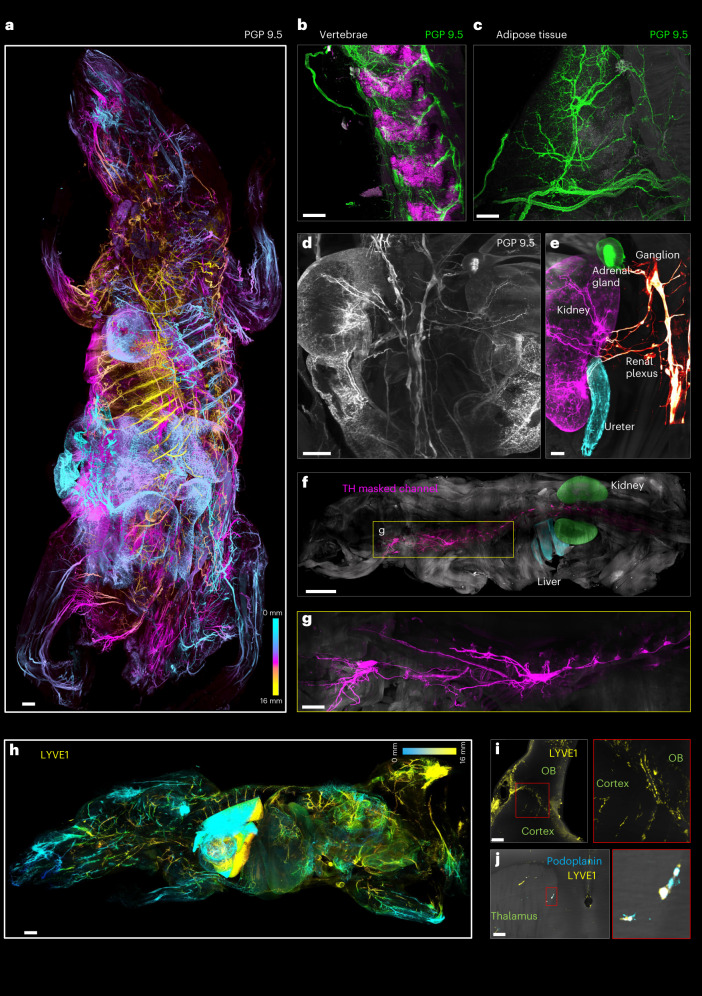
Comprehensive neuroanatomical and lymphatic mapping of the whole mouse body using wildDISCO. **a**, Depth color coding shows the pan-neuronal marker PGP 9.5^+^ neuronal projections at different *z* levels in the 2.0 cm-thick whole mouse body. Scale bar, 2,000 μm. **b**,**c**, Details of innervation throughout hard (vertebrae) (**b**) and soft tissues (adipose tissue) (**c**). Scale bars, 200 μm. **d**, Optical 2D section showed the PGP 9.5^+^ nerve innervation into multiple organs. Scale bar, 1,200 μm. **e**, Segmented vagus nerves innervating the kidney (magenta), adrenal gland (green), ureter (cyan), highlighted with specific pseudo-colors. Scale bar, 800 μm. **f**, Tracing of the TH^+^ vagus nerve over several organs. A single traced vagus nerve masked in magenta from the bottom of the spinal cord to the neck, kidney masked in green and liver masked in cyan. Scale bar, 4,000 μm. **g**, Higher magnification of the trajectories of the vagus nerve in the mouse can be determined. Scale bar, 1,500 μm. **h**, A whole mouse stained with a lymphatic vessel marker LYVE1 (yellow). Scale bar, 2,000 μm. **i**, Lymphoid elements (LYVE1) staining was detected in the brain parenchyma of the mouse. Scale bar, 150 μm. **j**, Mouse brains stained with two different lymphatic vessel markers (LYVE1 and podoplanin) to identify lymphatic endothelial cells found in the brain regions. Scale bar, 100 μm. **b**–**j**, *n* = 3.

**Extended Data Fig. 1:**
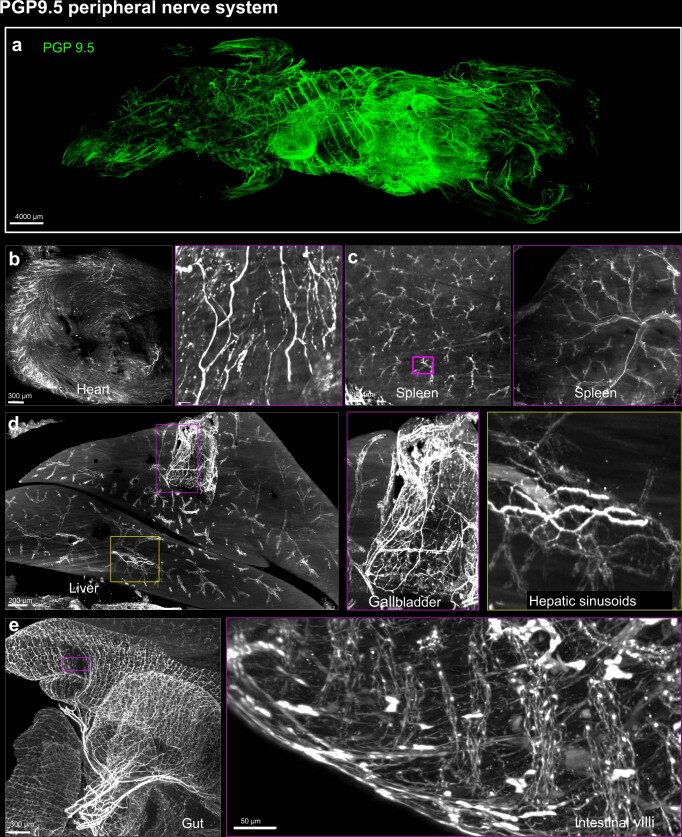
PGP9.5 peripheral nerve system. wildDISCO immunostaining of PGP9.5 throughout the mouse body (**a**) Maximum projection of the peripheral nervous system of a 4-week-old mouse stained with PGP 9.5 antibody using light sheet microscopy. (**b–e**) Examples of positive PGP 9.5 staining in different organs (heart, spleen, liver, and intestine) with more magnified areas. n = 3.

The sympathetic and parasympathetic systems are major parts of the autonomic nervous system that regulates and coordinates organ function. To provide a complete map of the innervation of organs in mice, we stained the peripheral neuronal network in young adult mouse bodies (roughly 4 weeks old, roughly 10 × 3 × 2 cm dimensions) using protein gene product 9.5 (PGP 9.5), a pan-neuronal marker, and imaged it at cellular resolution in its entirety using light-sheet microscopy (Fig. 2a, Extended Data Fig. 1 and Supplementary Video 1). The peripheral nerve system was homogeneously stained throughout the entire depth of the mouse body without apparent differences in signal intensity between tissues as different as vertebrae (Fig. 2b) and adipose tissue (Fig. 2c).

In the heart, for example, the network of nerve fibers coursing through the ventricular myocardium was evident (Extended Data Fig. 1b and Supplementary Video 2). The splenic parenchyma showed the nerve fibers’ complex, panicle-like architecture. The vagus nerve branched into smaller fiber bundles as it progressed toward the dorsal spleen, where we also visualized the splenic neural network (Extended Data Fig. 1c and Supplementary Video 3). Nerve fibers also innervated the hepatic sinusoids and distributed along the hepatic duct end-to-end. In the gallbladder, the ganglionated plexus comprising a series of irregularly shaped ganglia was clearly visible (Extended Data Fig. 1d and Supplementary Video 4). Whole mouse body tracing of nerves enabled us to visualize nerve connections between different organs (Fig. 2d,e and Supplementary Video 5), which will provide essential clues for understanding the role of nerve communication in normal physiology and disease.

**Extended Data Fig. 2:**
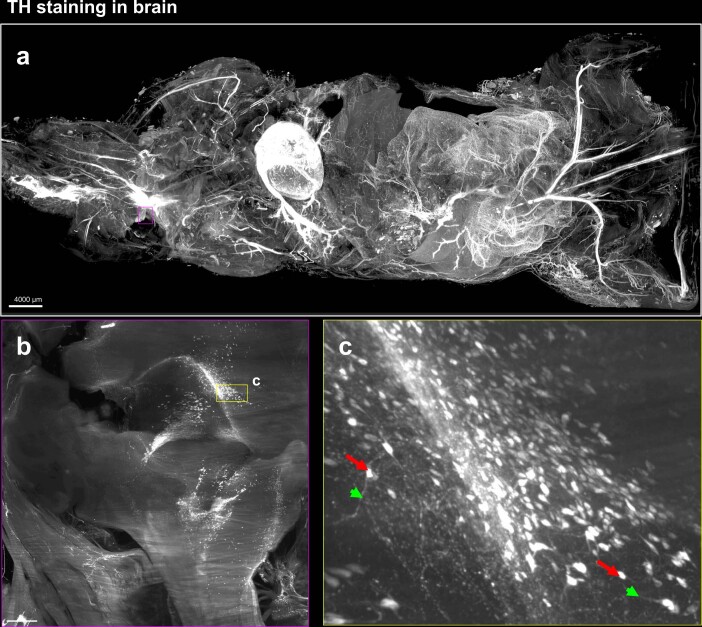
TH staining in brain. (**a**) Representative image of a 4-week-old mouse stained with TH antibody using lightsheet microscopy. (**b–c**) Examples of positive TH staining in the expected brain regions (**b**) with higher magnification areas showing TH-positive neuron axon (**c**), the red long arrow indicates neuron cell bodies, while the green short arrow indicates neuron filaments. n = 3.

Next, we assessed the sympathetic innervation of diverse organs using the sympathetic nerve marker TH. TH^+^ nerves were found throughout the body (Extended Data Fig. 2a), in most organs including the brain (Extended Data Fig. 2b,c) and visceral organs in 4-week-old mice. In the small intestine, the interconnected ganglionated plexuses on the intestinal wall were observed (Supplementary Video 6). We traced the vagus nerve (Fig. 2f,g and Supplementary Video 7) and showed that it provides sympathetic innervation to the abdominal organs and that it connects visceral organs, such as the kidneys, adrenal gland, ureter, liver, spleen and gastrointestinal tract. We obtained similar results in 3-month-old mice (Supplementary Fig. 9).

**Extended Data Fig. 3:**
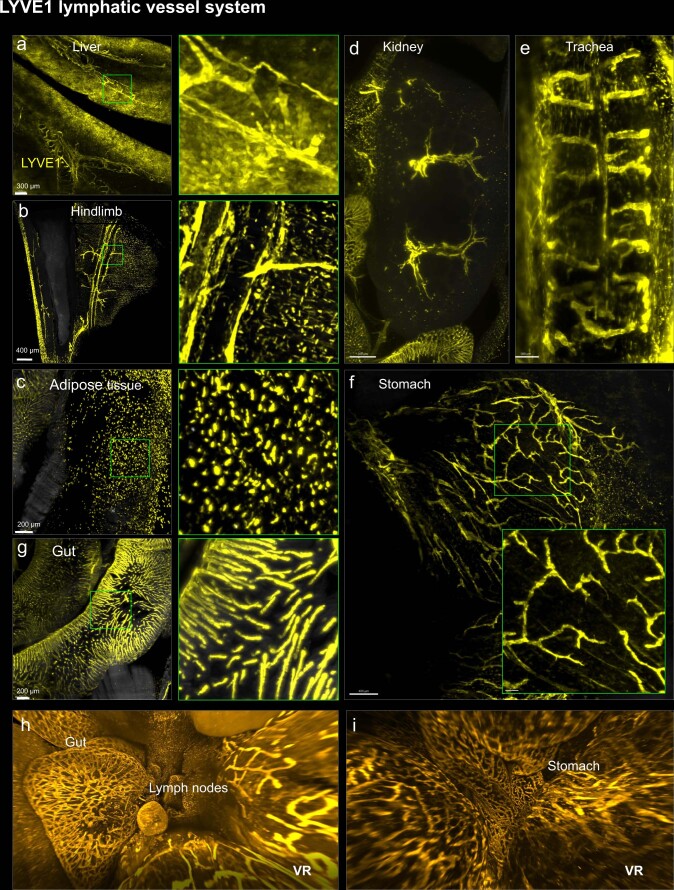
LYVE1 lymphatic vessel system. wildDISCO enables immunostaining of LYVE1 throughout the mouse body. Examples of optical sections of whole mouse staining with LYVE1 antibody in different organs, liver (**a**), hindlimbs (**b**), adipose tissue (**c**), kidney (**d**), trachea (**e**), stomach (**f**), and intestine (**g**) with higher magnified regions. (a-g), n = 3. (**h-i**) 3D reconstruction view of the intestinal lymphatic network using Syglass software.

To show the generalizability of the approach, we also mapped the lymphatic system across the mouse body using the lymphatic vessel endothelial hyaluronan receptor 1 (LYVE-1). We observed the finely structured lymphatic network throughout the body (Fig. 2h, Extended Data Fig. 3 and Supplementary Video 8) and could visualize details of lymph vessel organization in individual organs. For example, LYVE-1^+^ vessels were observed in the hepatic sinusoidal endothelium and the superficial gastrocnemius (Extended Data Fig. 3a,b). LYVE-1^+^ lymph nodes could be observed near the hindlimbs (Supplementary Video 9). Especially in adipose tissue, we observed a large variety in the shape and size of LYVE-1^+^ cells (Extended Data Fig. 3c). The larger lymphatic vessels of the kidney branched into lymphatic capillaries with a tree-like architecture (Extended Data Fig. 3d and Supplementary Video 10). Tracheal lymphatic vessels showed a segmental pattern of interconnected vessels (Extended Data Fig. 3e). In the stomach, lymphatics were unevenly distributed on the gastric walls and had tree-like branches (Extended Data Fig. 3f,i and Supplementary Video 11). Blunt-ended, tube-like lymphatic capillaries (lacteals)^21^ were clearly located in the intestinal villi (Supplementary Video 12), and the abundant and well-organized lymphatic plexuses and networks were visible on the outer surface of the intestinal wall (Extended Data Fig. 3g,h and Supplementary Video 13).

The brain parenchyma has been thought to be devoid of lymphatic vessels^22,23^, although there is lymphatic drainage from the central nervous system via meningeal lymphatic vessels^24^. Our whole-body immunolabeling data showed small and short lymphatic capillaries entering the brain parenchyma from the meninges. Some LYVE-1^+^ lymphatic vessels are also observed to connect the olfactory bulb with the cortex (Fig. 2i and Supplementary Video 14). These connections were observed by both LYVE1 and Prox1 (Prospero homeobox protein 1, a marker for lymphatic endothelium) staining. We also find lymph vessels entering the brain parenchyma around the thalamus (Supplementary Video 15), which was confirmed by both LYVE1 and podoplanin staining (Fig. 2j). Furthermore, we traced the LYVE1 lymphatic vessels from the brain to the vertebrae (Supplementary Video 16). We also extended LYVE1 labeling to mice with the skin. We could clearly visualize the network of lymphatic vessels in the skin. However, scanning of lymphatic vessels deep within internal organs while the skin was intact yielded poor-quality images. Imaging quality was restored when the skin was removed, and the mice were rescanned (Supplementary Fig. 10 and Supplementary Video 17).

**Extended Data Fig. 4:**
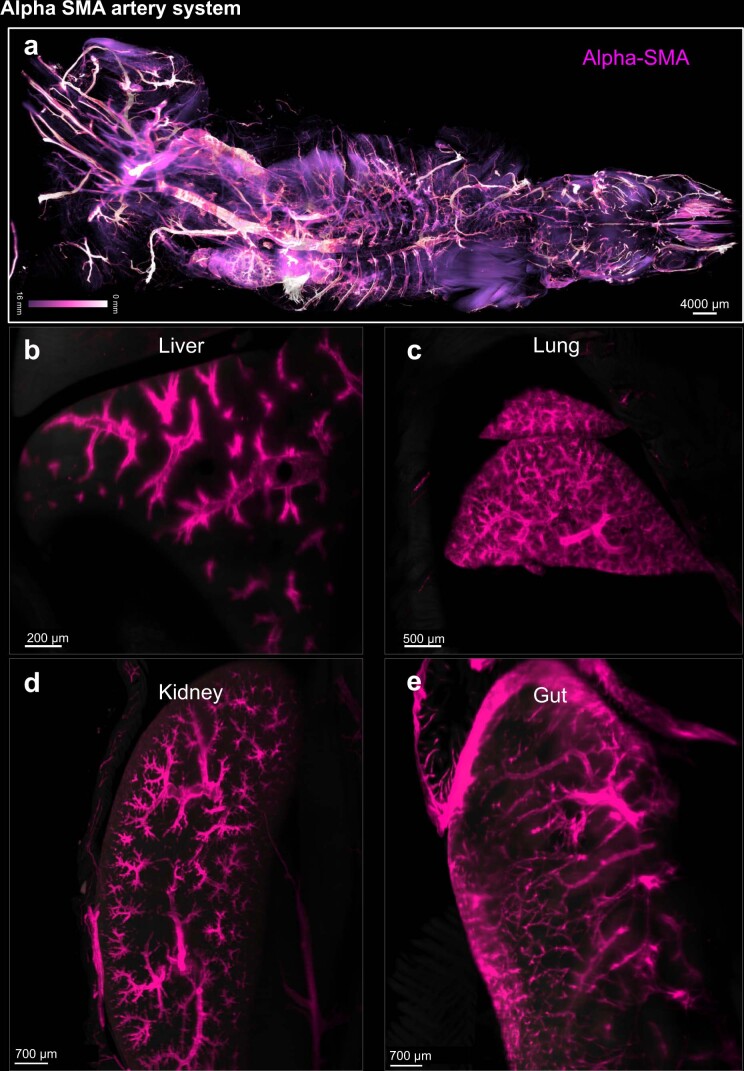
alpha-SMA artery system. (**a**) depth color map of the entire mouse body stained with alpha-SMA. Examples of optical sections of whole mouse staining with alpha-SMA antibody in different organs, liver (**b**), lung (**c**), kidney (**d**), and gut (**e**). n = 3.

**Extended Data Fig. 5:**
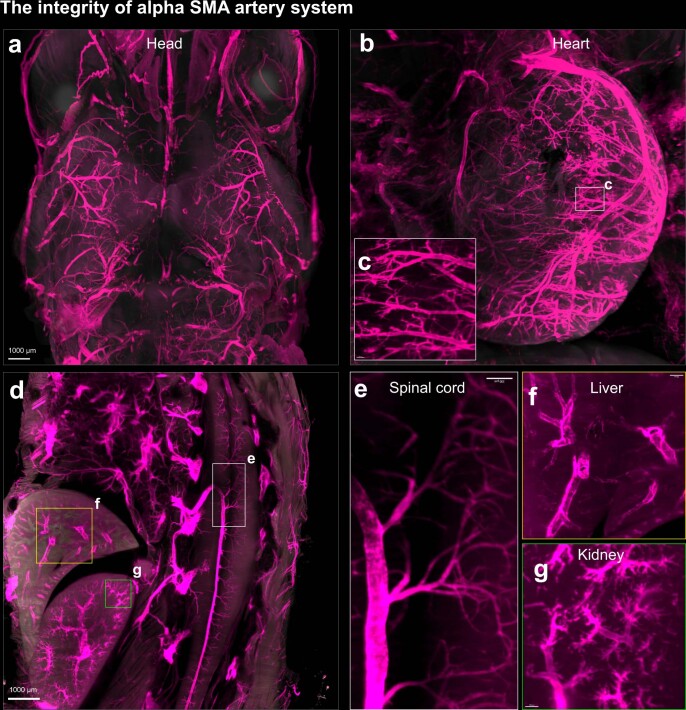
The integrity of alpha SMA artery system. (**a**) Examples of optical sections of whole mice stained with alpha-SMA to show the integrity of the vasculature in various organs: head (**a**), heart (**b**), higher magnification of heart regions (**c**), multiple connected internal organs (**d**) with higher magnification regions: spinal cord (**e**), liver (**f**), and kidney (**g**). n = 3.

Next, we used wildDISCO to generate a body-wide map of arteries. We used alpha-SMA as an arterial marker (Extended Data Fig. 4), and observed continuous arteries in multiple organs, including the brain, liver, spleen, heart and spinal cord. Particularly in the head, in the heart and near the spinal cord (Extended Data Fig. 5), we clearly observed the blood vessel connections and proved the integrity of vasculature staining.

### wildDISCO is compatible with double labeling

Next, we investigated whether wildDISCO can be used to label more than one protein in the same sample, as this would allow us to study the relationship of different physiological systems using conventional antibodies.

**Fig. 3:**
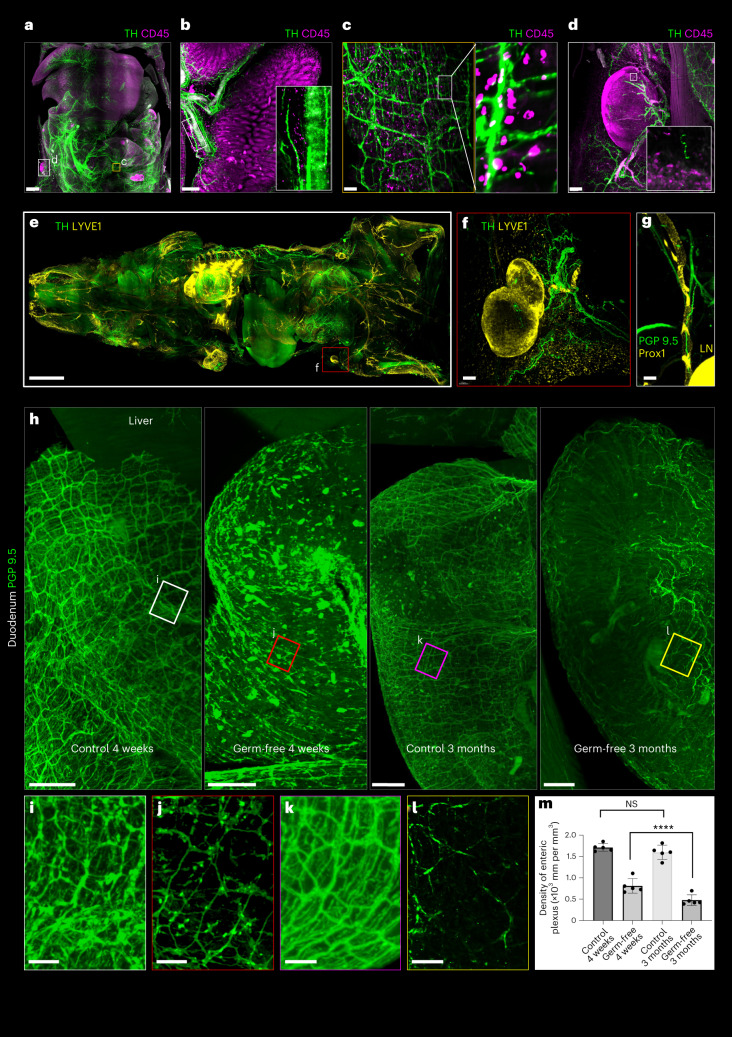
Different physiological system staining using wildDISCO. **a**, Maximum intensity projection of a mouse stained with antibodies against the TH (green) and the immune cell marker CD45 (magenta), showing the landscape of neuro-immune interactions in internal organs. Scale bar, 1,000 μm. **b**, The branches of the sympathetic nervous system (TH, green) connect different regions of the intestine. CD45^+^ cells (magenta) accumulate along parts of the sympathetic nerve, especially at the inferior mesenteric plexus. Scale bar, 200 μm. **c**, High-magnification views of the labeled regions in **a**, showing the colocalization of the sympathetic nerve fibers and immune cells on the intestinal wall. Scale bar, 200 μm. **d**, Representative 2D optical sections of peripheral nerves with immunomodulatory in lymph node (LN) stained with TH and CD45. Scale bar, 100 μm. **e**, Maximum intensity projections of a whole mouse stained with TH (green) and LYVE1 (yellow). Scale bar, 3,000 μm. **f**,**g**, Representative 2D optical sections of hindlimb LNs stained with TH and LYVE1 (**f**) and PGP 9.5 and Prox1 (**g**) as indicated in the images to show the LNs are innervated by peripheral nerves with immunomodulatory potential. Scale bars, 150 μm. **h**, 3D representation of the enteric nerve lattice network of wildtype mice and germ-free mice by immunostaining with antibodies against PGP 9.5. Scale bars, 500 μm. **i**–**l**, Higher-magnification views of the regions marked by the white (**i**), red (**j**), magenta (**k**) and yellow (**l**) boxes. Scale bars, 300 μm. **a**–**l**, *n* = 3. In the germ-free mice, the enteric nerve lattice network appears disorganized, with fewer ganglia. **m**, The density of the PGP 9.5 enteric plexus was quantified. *n* = 5; mean ± s.d.; ****P = 3.27 × 10^−10^, NS, *P* > 0.05 (one-way analysis of variance). Source data

**Extended Data Fig. 6:**
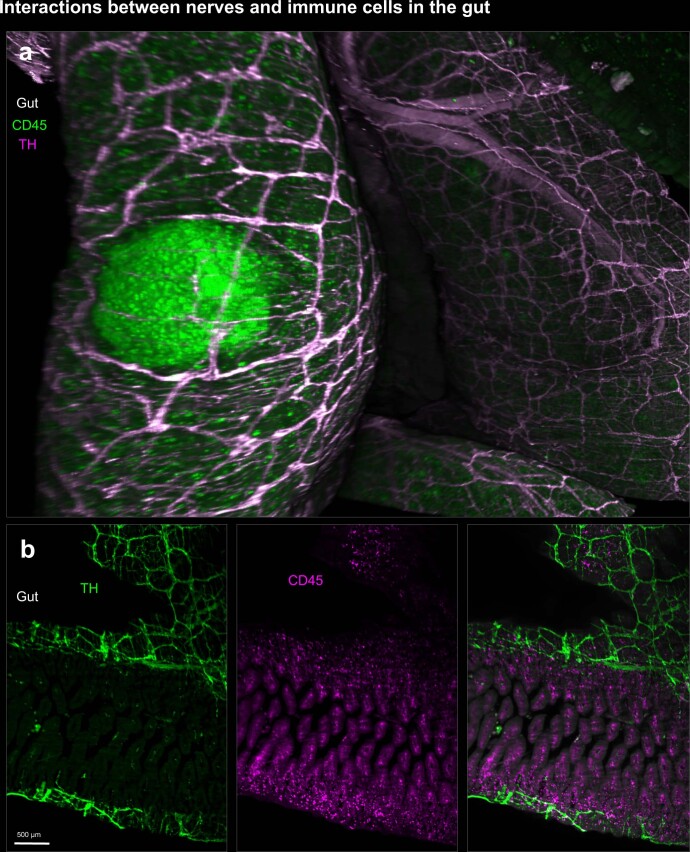
Interactions between nerves and immune cells in the gut. (**a**) 3D reconstruction view of a Peyer’s patch in the gut stained with CD45 (green) and innervated by TH + sympathetic nerve (magenta) visualized with Syglass software. (**b**) Sympathetic nerve marker TH (green) and immune cell marker CD45 (magenta) on the intestinal wall visualized with Imaris software.

**Extended Data Fig. 7:**
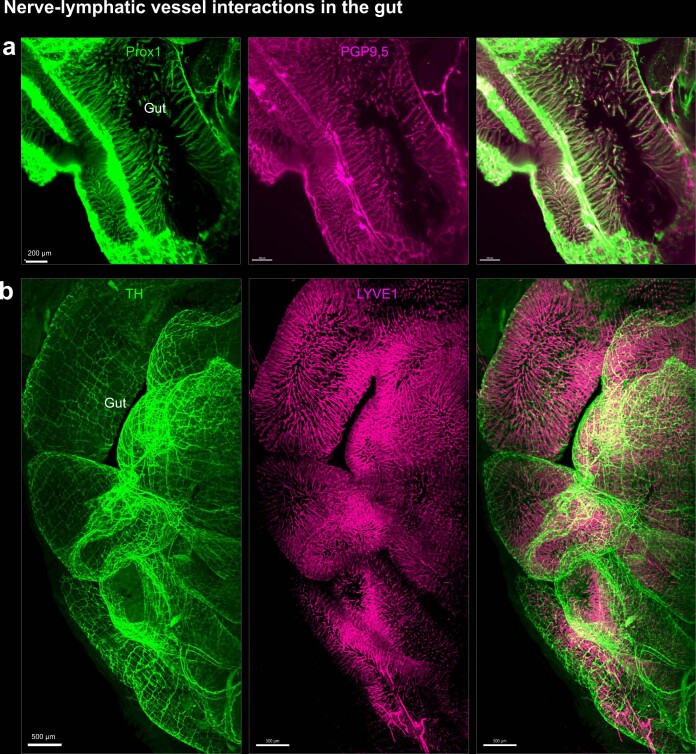
Nerve-lymphatic vessel interactions in the gut. (**a**) PGP9.5 nerve fibers (magenta) interacted with Prox1 lymphatic vessel (green). (**b**) TH sympathetic nerve (green) and LYVE1 lymphatic vessel (magenta). n = 3.

First, we coimmunolabeled TH^+^ sympathetic nerves and CD45^+^ immune cells (Fig. 3a–d, Extended Data Fig. 6 and Supplementary Video 18). We find substantial colocalization of immune cells along parts of the sympathetic nervous system, especially at the inferior mesenteric plexus (Fig. 3b), and frequent contacts between immune cells and sympathetic nerves on the intestinal wall (Fig. 3c,d). To better illustrate neuro-immune interactions in the lymphatic system, especially the lymph nodes, we used double staining of nerve fibers and lymphatic vessels (Fig. 3e, Extended Data Fig. 7, Supplementary Fig. 11 and Supplementary Videos 19–22). LYVE1^+^ and Prox1^+^ lymph nodes were often innervated with TH^+^ (sympathetic neuronal marker) or PGP 9.5^+^ (pan-neuronal marker) neuronal processes (Fig. 3f,g and Supplementary Videos 23 and 24). Double staining of nerve and lymphatic cells, as well as nerve and immune cells, revealed the intricate interactions of these systems throughout the body.

### wildDISCO reveals neuronal abnormalities in germ-free mice

**Extended Data Fig. 8:**
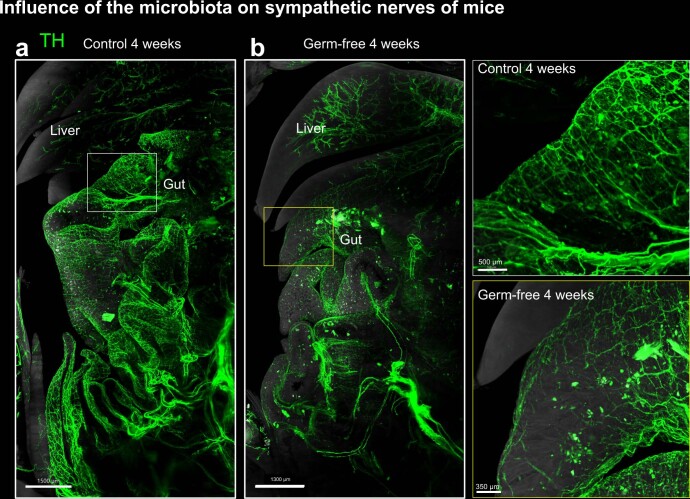
Influence of the microbiota on sympathetic nerves of mice. (**a–b**) The enteric nerve lattice network of WT mice and germ-free mice by immunostaining with antibodies against TH. Higher magnification views of the regions marked by the white and yellow boxes, respectively. n = 3.

Next, we used wildDISCO to study perturbations of biological systems at whole mouse body level. Microbiota, which are the collection of microorganisms that live in and on the body, have been suggested to play an important role in the development and function of the nervous system^25^. For example, germ-free mice have been shown to have altered behavior, including increased anxiety-like behavior and impaired social behavior^26,27^. Although these behavioral changes may be related to alterations in neural development and function that occur in the absence of microbiota, the extent of the structural abnormalities across the whole body remains to be explored. Comparing the structure of the nervous system of germ-free mice and wildtype controls, we found that the PGP 9.5+ nerve lattice network in the intestinal wall was substantially less dense in 4-week-old germ-free mice compared to same aged wildtype controls (Fig. 3h and Extended Data Fig. 8). In 3-month-old germ-free mice, axons were even more degenerated and the lattice network less dense than in 4-week-old germ-free mice (Fig. 3i–l). These findings suggest that the microbiome plays a role in axon development and innervation of peripheral body regions^28^ throughout different ages of the mice.

### Revealing tertiary lymphoid structures and rare cells

Next, we investigated rare structures called tertiary lymphoid structures (TLS) in tumor-bearing mice. TLS are organized aggregates of immune cells that form in non-lymphoid tissues. TLS are not found under physiological conditions but arise in the context of autoimmune disease and cancer. For example, TLS can promote infiltration of immune cells into the tumor site. They also attracted interest as a means of enhancing anticancer immunity^29^. However, how TLS are distributed throughout the body in relation to the primary tumor and metastasis has been unclear.

**Fig. 4:**
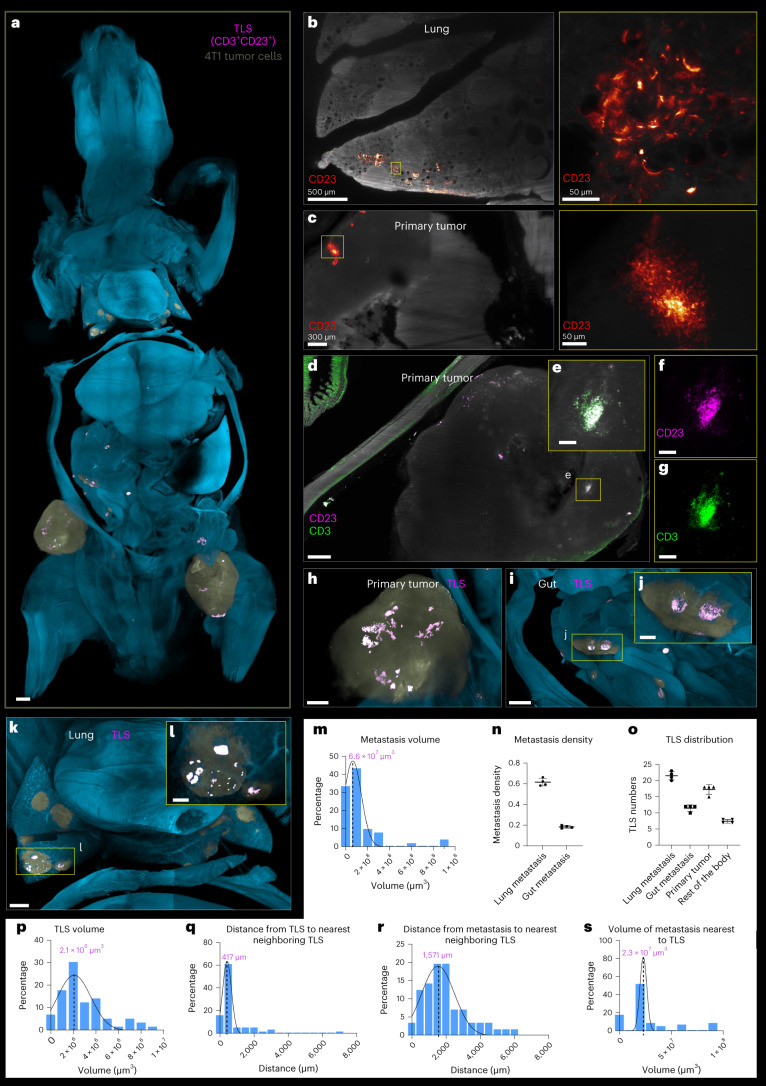
Visualization and analysis of tumor-associated TLS in a tumor metastasis model using wildDISCO and Deep Learning. **a**, 3D rendering of a mouse with 4T1 cell metastases using light-sheet microscopy imaging in ventral view. The TLS are detected and masked in magenta, the tumor cells masked in yellow and the background color is cyan. A higher magnification view shows details of the TLS. Scale bar, 2,000 μm. **b**–**l**, Example images of TLS in a mouse with tumors, stained with CD23 in red (**b,c**) and CD3 in green and CD23 in magenta (**d**–**g**). TLS masked in magenta in the primary tumor (**h**), gut (**i,j**) and lung (**k**,**l**). Scale bars: 500 μm (**d**,**h**,**k**); 150 μm (**e**,**f**,**g**,**j**); 400 μm (**i**) and 200 μm (**l**). **m**–**s**, Quantification of the spatial correlation between TLS and metastases throughout the mouse. **m**, Quantification of the metastasis volume across the mouse. **n**, Quantification of the metastasis density in lung and gut. *n* = 4 mice. mean ± s.d. **o**, Quantification of the distribution of TLS throughout the mouse. *n* = 4 mice. mean ± s.d. **p**, Quantification of the TLS volume across the mouse. **q**,**r**, Quantification of the distance to nearest neighboring TLS (**q**), and between metastases and the nearest TLS (**r**). **s**, The metastasis volume to the nearest TLS. Source data

**Extended Data Fig. 9:**
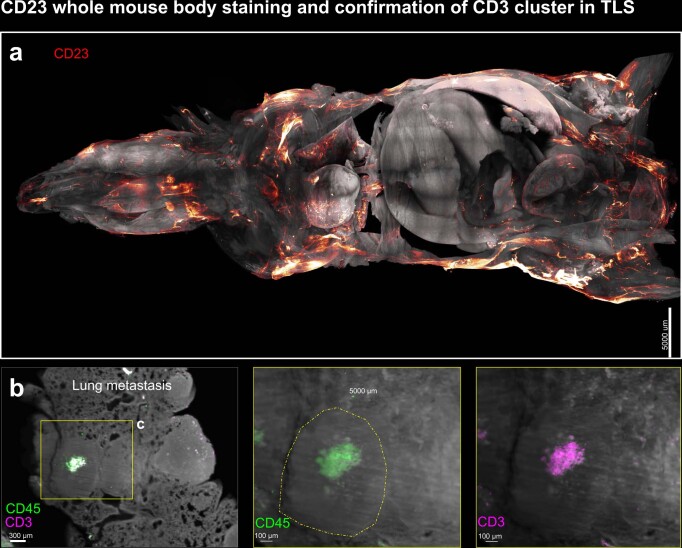
CD23 whole mouse body staining and confirmation of CD3 cluster in TLS structure. (**a**) wildDISCO immunostaining of mature TLS marker CD23 in the whole mouse body of 4T1 cancer metastasis. (**b**) Representative 2D optical image of lung metastasis with immune cell marker CD45 in red and T cell marker CD3 in magenta to confirm TLS structure. n = 3.

To visualize TLS in the whole body with cancer metastasis, BALB/c mice were implanted with 4T1 breast cancer cells subcutaneously. After 14 days, we euthanized the mice and used wildDISCO to stain TLS using CD23 (conventional B cells and follicular dendritic cells marker) and CD3 (a T cell marker) antibodies (Fig. 4a, Extended Data Fig. 9a and Supplementary Video 25). We also demonstrated the specific signal of CD3^+^ T cells using costaining of total immune cells with CD45 (Extended Data Fig. 9b). We find conventional B cells, follicular dendritic cells and T cells clusters and/or aggregates (denoting TLS) in the primary tumor (Fig. 4c–h) and in some metastatic sites, such as those in the lung and gut (Fig. 4b,i–l). Notably, there are no suitable reporter mice (such as CD23^+^) that can label TLS. This makes wildDISCO uniquely capable of investigating TLS and their distribution in whole mice.

We analyzed the size and distribution of TLS throughout the body in association with cancer metastasis. Micrometastases were randomly distributed throughout the mouse body, regardless of their volume, suggesting independent colonization at multiple sites by this tumor model. More than 80% of micrometastases were within 0.02 mm^3^ (Fig. 4m), mainly distributed in the lung (62%) and gut (19%) (Fig. 4n). However, the spatial correlation between formation of TLS and nearby cancer metastases at the whole-body level is not clearly known. First, we quantified the volume and relative location of all TLS in the whole body. The TLS were mainly located near the lung and gut metastases, and their density was positively correlated with the metastasis density (Fig. 4o). The volume of TLS was notably smaller in comparison to that of the metastases; even large TLS (average volume 0.0021 mm^3^) were roughly 30 times smaller than metastases (average volume 0.066 mm^3^) (Fig. 4p versus Fig. 4m). Given their smaller size, detecting TLS at the whole-body level using alternative methods presents a challenge. To better comprehend the spatial distribution of TLS and metastases across the entire mouse, we made several observations. First, individual TLS were often separated by a specific distance: the smallest distance between most of TLS and other TLS being average roughly 0.4 mm (Fig. 4q). Second, we noted a significant discrepancy between the distances of metastases and their closest TLS, with a majority average measurement of roughly 1,571 mm (Fig. 4r). Last, we found that the average volume of metastases to the nearest TLS is 0.023 mm^3^, smaller than the average metastasis size throughout the body (Fig. 4s versus Fig. 4m). These insights enrich our understanding of the characteristics and interactions between TLS and metastases within the studied mouse model.

**Extended Data Fig. 10:**
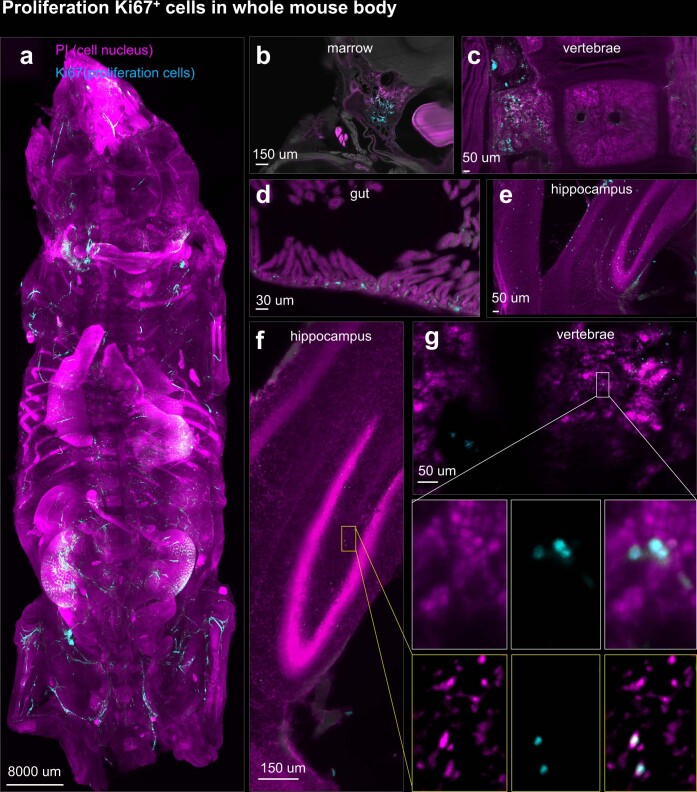
Proliferation Ki67+ cells in whole mouse body. (**a**) wildDISCO immunostaining of cell proliferation marker Ki67 (cyan) and PI (magenta) in the whole mouse. (**b**) Representative 2D optical image of Ki67-positive proliferation cells (cyan) as rare cells compared to nuclear staining (propidium iodide, PI) in the bone marrow (b), vertebrate (**c**), gut (**d**) and hippocampus (**e**). (**f–g**) Single proliferation cells identified in brain (f) and vertebrate (g) by whole-body light-sheet microscopy scans. Higher magnification images show colocalization of proliferation cells with a single nucleus. n = 3.

Next, we used wildDISCO to study the distribution of rare proliferating cells throughout the mouse using Ki67 antibody^30^ labeling (Extended Data Fig. 10a). As expected, we found proliferating cells in bone marrow niches (Extended Data Fig. 10b,c), at the base of gut crypts (Extended Data Fig. 10d) and in the dentate gyrus of the hippocampus (Extended Data Fig. 10e,f). High-magnification images confirmed the specificity of the signal at the single-cell level by colocalization with the cell nucleus, as indicated by propidium iodide staining (enlarged boxes in Extended Data Fig. 10f,g). We further observed Ki67^+^ cells in more unexpected body regions including in the spinal cord (Supplementary Videos 26 and 27). Thus, wildDISCO can provide insight into the distribution of rare cells, including proliferating cells throughout the mouse body.

### wildDISCO generated online whole mouse atlases

**Fig. 5:**
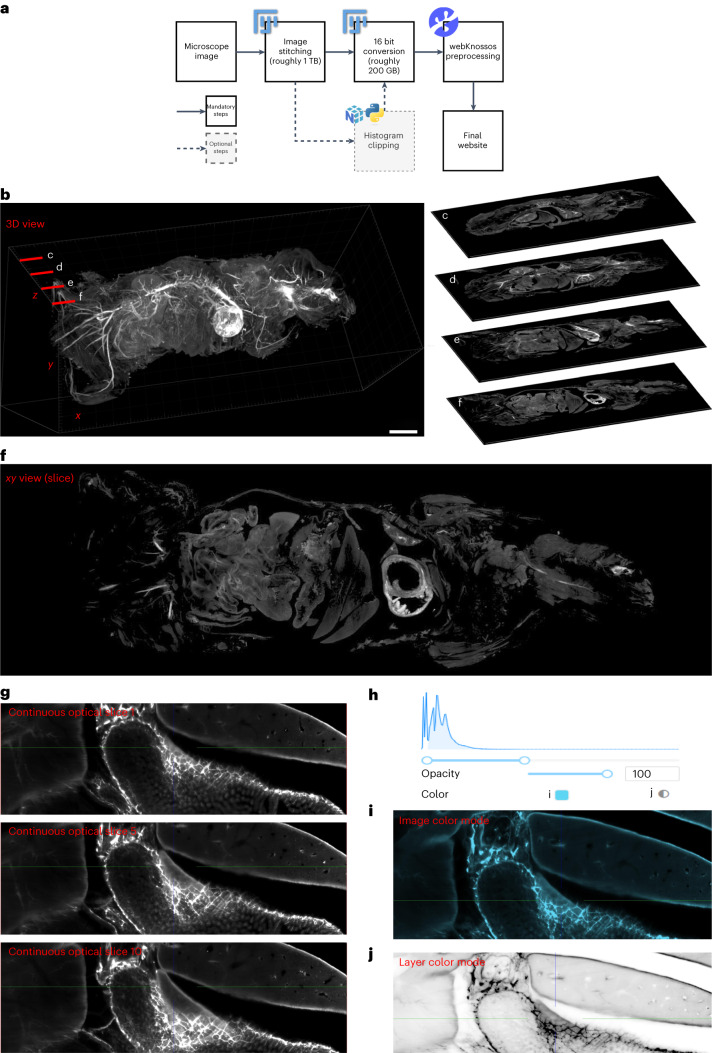
Whole mouse body atlas website and its features. **a**, Flowchart outlining the process of creating a whole mouse atlas website. **b**–**f**, The representative sections of a whole-body atlas in a 3D view. Scale bar, 5,000 μm (**b**). The images represent exemplary slices of an entire mouse taken at different depths and viewed from a dorsal perspective. For example, slices contain the spinal cord (**c**), the liver and the lung (**d**), the gut and the heart (**e**) and the spleen (**f**). **g**, Continued optical sections and a representative image of the continued view from slices 1 to 10. **h**–**j**, Different display models. **h**, Opacity and color. **i**,**j**, Selection of cyan image in image color mode (**i**) and selection of monochrome image in layer color mode (**j**).

After generating high-resolution images of whole mouse systems, such as the nervous system, lymphatic system and vascular system, we aimed to make these data available to the scientific community in online atlases. To this end, we established a website (Fig. 5) (http://discotechnologies.org/wildDISCO/atlas/index.php), which allows researchers to explore our reference datasets. We provided a tutorial video (Supplementary Video 28). This website offers different views of the entire mouse (*xy*, *xz*, *yz* planes). Compared to the traditional histology sections, our whole mouse atlas provides continuous optical slices without skipping tissue regions (Fig. 5b–g). The website also offers online contrast and color adjustments (Fig. 5h–j). We envisage that this online tool will prove invaluable for biomedical research, allowing scientists to examine these systems in whole mice without the need to replicate the same experiments.

## Discussion

We have developed a whole-body IgG antibody immunolabeling method called wildDISCO (Supplementary Video 29). The CD5-based tissue permeabilization chemistry permits uniform staining of whole bodies with conventional (150 kDa size) IgG antibodies, producing whole mouse body cellular and structural maps akin to the Allen Brain Atlas. Previously published whole mouse body clearing and imaging techniques heavily relied on transgenic reporters or small dye labeling^5,31–34^, which were incompatible with the thousands of commercially available IgG antibodies, limiting their applications.

We systematically compared wildDISCO with existing clearing methods, including vDISCO^5^, iDISCO^11^, uDISCO^31^ and PEGASOS^32^, demonstrating the advantages of our technology. By using wildDISCO, we uniformly labeled and imaged various biological systems throughout entire mouse bodies, such as the nervous system, lymphatic vessels, blood vasculature, immune cells and proliferating cells.

We also showed that wildDISCO can facilitate the analysis of pathogenic processes by comparing neuronal abnormalities in germ-free mice (at 4 weeks and 3 months old) and mapping TLS in cancer metastasis. As modulators of tumor immune responses, TLS are of high interest in this age of tumor immunotherapy. It has been shown that the presence and characteristics of TLS in the tumor microenvironment can influence the prognosis and response to therapy in various types of cancer. In several types of cancer, the presence of TLS has been associated with improved survival and lower rates of recurrence^35^. Thus, there is a growing interest in developing therapeutic strategies to modulate TLS formation and function to enhance immune responses against cancer^36^. However, the exact mechanisms and implications of TLS in cancer are still not completely understood. Here we presented a tool to investigate TLS at the whole mouse body level using wildDISCO, which should be of interest to the wider scientific community beyond cancer as TLS play critical roles in many diseases including autoimmune diseases such as rheumatoid arthritis and Sjögren’s syndrome^37^, atherosclerosis^38^ and multiple sclerosis^39^.

A benefit of wildDISCO is that we do not need to rely on transgenic reporters to assess proteins at the systems level. Transgenic reporter lines are in general expensive to develop and maintain, and in many cases impossible to generate. Additionally, breeding and crossing such reporter lines with the disease models is time consuming. Furthermore, such transgenic mice are limited to specific mouse strains, such as only C57BL/6 or CD-1 IGS, making it difficult to cross disease models from other backgrounds. WildDISCO overcomes these hurdles as the distribution of proteins can now be investigated in any mouse line and disease model using whole mouse IgG labeling.

Although wildDISCO has proved successful with a wide range of IgG antibodies, further validation with a broader selection of antibodies is necessary. Here, we have already listed more than 30 validated antibodies, and we will continue to update the validated antibodies regularly for the scientific community at the online website (http://discotechnologies.org/wildDISCO/). Second, the high-resolution three-dimensional (3D) imaging generated by wildDISCO results in large amounts of data that can be challenging to analyze effectively. The development of specific deep learning algorithms tailored to handle such datasets is needed for automating and streamlining the analysis process, which will enable researchers to extract valuable insights from the data more efficiently. Also, sharing terabytes of mouse atlases online is currently a challenge due to the lack of appropriate infrastructure in most research institutes. To foster collaboration and enable researchers to build on the findings of others, it is vital to develop and implement robust data sharing platforms that can handle the large-scale data generated by wildDISCO and similar imaging techniques.

In summary, wildDISCO is a whole-body IgG antibody immunolabeling method that allows researchers to obtain comprehensive cellular and structural maps of entire mouse bodies. Aided by the virtual reality (VR) visualization, previously inaccessible 3D anatomical information becomes possible (for example, see Supplementary Videos 30 and 31). By facilitating the use of off-the-shelf IgG antibodies, wildDISCO broadens the applicability of whole-body clearing and imaging techniques. Our presentation of whole mouse body atlases will enable a deeper understanding of complex biological systems. The insights gained from this technology have the potential to enhance our knowledge of disease initiation, progression and extent at the whole organism level, ultimately contributing to the development of more effective diagnostic and therapeutic strategies.

## Methods

### Animals involved in the study

We used the following mix-gender animals for the wildDISCO study: 4-week-old, 6-week-old and 3-month-old wildtype mix-gender mice (C57BL/6J, CD-1 IGS and Balb/c) purchased from Charles River Laboratories. Animals were housed on a 12/12 h light/dark cycle and had random access to food and water. Temperature was maintained at 18–23 °C and humidity was at 40–60%. The 4-week-old and 3-month-old C57BL/6J germ-free mix-gender mice were purchased from the Technical University of Munich (Institute of Nutrition and Health, Core Facility Gnotobiology), and were housed in a germ-free isolator house. The absence of bacteria was confirmed in the germ-free mice by microbial cultures, and mice were then used for further experiments. Each antibody was repeated successfully on at least five mice and also by at least three different people. Animal experiments were performed according to the institutional guidelines of the Ludwig Maximilian University of Munich and the Helmholtz Munich Center German Mouse Clinic after approval of the Ethical Review Board of the Government of Upper Bavaria (Regierung von Oberbayern, Munich, Germany).

### Cyclodextrin buffer screening for cholesterol extraction

Cholesterol extraction was measured using the cholesterol/cholesterol ester-GloTM assay (Promega). Here, 25 mg of paraformaldehyde (PFA) fixed mouse liver was incubated in 3 ml of 1% w/v different cyclodextrin-containing antibody buffers: 2-hydroxypropyl-β-cyclodextrin (PanReac AppliChem, A0367,0100), methyl-β-cyclodextrin (Sigma-Aldrich, 332615-25G), (2-hydroxyethyl)-β-cyclodextrin (Sigma-Aldrich, 389137-10G), triacetyl-β-cyclodextrin (Sigma-Aldrich, 332623-10G), succinyl-β-cyclodextrin (Sigma-Aldrich, 85990-500MG) and heptakis(2,6-di-*O*-methyl)-β-cyclodextrin (Sigma-Aldrich, 39915-1G). The assays were measured at different time points (2, 3, 5 and 7 d). Then, a 5 μl aliquot of the supernatant was diluted tenfold in cholesterol lysis solution and incubated for 30 min at 37 °C. Cholesterol detection reagent was then added to the samples and incubated for 60 min at room temperature. The value was measured using a Centro LB 96-plate reading luminometer (Berthold).

### Cyclodextrin buffers’ impact on antibody stabilization

The homogeneity of antibodies, in other words of antibody aggregations, in different cyclodextrin-containing buffers was measured by DLS. TH primary antibody was selected to evaluate antibody stabilization and homogeneity. TH antibody (Millipore, AB152) (molecular weight 150 kDa, concentration 10 g l^−1^) was dissolved in HEPES buffer (4-(2-hydroxyethyl)-1-piperazineethanesulfonic acid) with and without CD5 (Sigma-Aldrich, 39915-1G) (1% w/v) at room temperature. After 7 days of incubation, the buffer solutions were diluted and afterward measured in a folded capillary cell (DTS 1070) using a Zetasizer Nano ZS. Samples were measured three times with six sub-runs each. The temperature was set to 25 °C.

### Permeabilization of brain with cyclodextrin buffers

Mouse half brains were incubated with various cyclodextrin buffers at 1% (w/v), 45 ml for 3 days at 37 °C. After washing twice with PBS, the samples had 45 ml of 0.03% methylene blue added to them and were incubated overnight at 37 °C.

To determine the efficiency of methylene blue staining after incubation with different cyclodextrin buffers, samples were cut in half along the middle line to evaluate the efficacy of the inner tissue staining. The methylene blue-stained mouse brains were cryosectioned into 10 μm slices for higher resolution images using Zeiss Axio Imager.M2 microscopy. The images of the samples were analyzed by ImageJ for profile plot along and the pixels were quantified under threshold gray value.

### Breast cancer metastasis model

Some 4T1 breast cancer cells, encoded with EGFP and enhanced firefly luciferase, were filtered through a 100 μm membrane and resuspended in RPMI 1640 medium (GIBCO, 11875093). For the subcutaneous injection model, 1 × 10^6^ cancer cells (50 μl) were injected into the fourth left and right mammary fat pads of 6-week-old female Balb/c mice. After 14 days, the metastases of mice were measured by bioluminescence using IVIS Lumina II Imaging System (Caliper Life Sciences). Briefly, the mice were anesthetized with ketamine, fixed in the imaging chamber and imaged 15 min after injection of luciferin (150 mg kg^−1^, intraperitoneal injection). The bioluminescence signal was quantified using Living Image Software v.4.2 (Caliper Life Sciences). After confirmation of metastasis occurrence by IVIS imaging, the mice were euthanized and stained with two antibodies CD3 and CD23 to confirm TLS.

### Perfusion and whole mouse body fixation

Mice were deeply anesthetized with MMF (0.05 mg kg^−1^ fentanyl, 0.5 mg kg^−1^ medetomidine and 5 mg kg^−1^ midazolam with intraperitoneal injection) and perfused intracardially with heparinized 0.01 M PBS (10–25 U ml^−1^ final heparin concentration, Ratiopharm, N68542.03; perfusion volume 12 ml min^−1^ with an ISMATEC peristaltic pump system). After washing the blood out of the mice for 5–10 min, 4% PFA in 0.01 M PBS (Morphisto, 11762.01000) was perfused 10–20 min. The mouse bodies were skinned and transferred to 0.01 M PBS after postfixation in 4% PFA for 6 h at 4 °C.

### Whole-body staining with wildDISCO protocol

The wildDISCO whole-body immunostaining protocol is mainly based on a setup for pumping the pretreatment solutions and immunostaining buffers through the mouse heart and vasculature to perfuse the whole body. The pumping setup has been previously described^6,13^. In brief, after postfixation of PFA and 0.1 M PBS washing twice for 30 min, the mouse body was placed in a 300 ml glass chamber and the perfusion needle was inserted into the mouse heart through the same hole as in PFA perfusion. Then, the perfusion needle was connected to an ISMATEC peristaltic pump (REGLO Digital MS −4/8 ISM 834; reference tube, SC0266), which maintained pressure at 160–230 mmHg (45–60 rpm) and was used to establish transcardiac circulation. The pump was equipped with two channels. One was used to pump the solution through the heart to circulate throughout the mouse, while the second channel collected and circulated the solution leaving the mouse body. In the first channel, a 1-ml syringe tip (Braun, 9166017V) was used to connect the perfusion needle (Leica, 39471024) and the reference tube (Ismatec Reglo, SC0266), which is from the pump and set for circulation of the solution through the heart into the vasculature. Since the second channel allowed the solutions to recirculate, the inflow tubing was immersed in the solution chamber of the glass chamber. After the pump and channels are set up, the needle tip was fixed with superglue (Pattex, PSK1C) to ensure continuing and stable perfusion. All the following perfusion steps were performed using the setup explained above. The mice were first perfused with 0.1 M PBS overnight at room temperature, followed by 2 days of perfusion with the decalcification solution containing 10 w/v% EDTA (Carl Roth, 1702922685) in 0.1 M PBS, and the pH was adjusted to 8–9 with sodium hydroxide (Sigma-Aldrich, 71687) to decalcify all bones at room temperature. Then, the mouse bodies were perfused three times with 0.1 M PBS and washed for 3 h each time. Next, each mouse was perfused for 1 day with permeabilization and blocking solution containing 10% goat serum and 2% Triton X-100 in 0.1 M PBS. Next, the mice bodies were perfused with primary antibodies (antibodies are listed in Supplementary Table 1; validated antibody list http://discotechnologies.org/wildDISCO/) and incubated for 7 days with 250 ml of immunostaining buffer containing 3% goat serum, 10% CHAPS, 2% Triton X-100, 10% dimethylsulfoxide (DMSO), 1% glycine, 1% CD5 in 0.1 M PBS. Each mouse body was then washed three times in 0.1 M PBS and each time took 12 h at room temperature. Then, the mice bodies were perfused in the immunostaining buffer at room temperature with the Alexa fluorescent dye-conjugated secondary antibodies: Alexa Fluor 647 goat antirabbit IgG antibody (Thermo Fisher Scientific, A-21245) or Alexa Fluor 647 goat antirat IgG antibody (Thermo Fisher Scientific, A-21247) (25 µg in 250 ml, diluted 1:10,000) for 7 days. The mice bodies were washed three times with 0.1 M PBS, each time for 12 h. After the immunostaining steps were done, the mice were transferred to a fume hood and were cleared using the 3DISCO passive whole-body clearing protocol as previously reported^9^. Briefly, mice bodies were placed in a 300 ml glass chamber and immersed in 200 ml of the following gradient of tetrahydrofuran (Roth, CP82.1) in distilled water with gentle shaking (50% once, 70% once, 80% once, 100% twice, 12 h for each step), followed by 3 h in dichloromethane (Sigma, 270997) and finally in benzyl alcohol and benzyl benzoate (ratio of 1:2, Sigma, 24122 and W213802) until the bodies were optically transparent.

### Comparison of different whole-body clearing methods

The detailed protocol of vDISCO was described previously^5^. However, all previously used nanoboosters were replaced with commercial IgG antibodies at a concentration of 25 µg per mouse. For the iDISCO antibody staining method, we followed the original iDISCO+ publication^11^ and the latest protocol updates from https://idisco.info. Briefly, each step was adjusted to the whole-body level instead of the organ-level (for example, the duration of methanol dehydration was increased to 6 h). After pretreatment with methanol, the entire body was treated with a series of iDISCO+ solutions. For immunolabeling, the whole mouse was treated with the primary antibody (Synapsin-1, CST, no. 5297, 25 µg in 250 ml, diluted 1:10,000) in the first solution (0.2% Tween-20, 5% DMSO, 3% donkey serum, 1 mg l^−1^ heparin in PBS) at 37 °C for 7 days and then incubated with a secondary antibody (Alexa Fluor 647 goat antirabbit IgG antibody, Thermo Fisher Scientific, A-21245, 25 µg in 250 ml, diluted 1:10,000) in the second solution (0.2% Tween-20, 5% DMSO, 3% donkey serum, 1 mg l^−1^ in PBS) at 37 °C for 7 days. Finally, the whole mouse was cleared until it became optically transparent. For the uDISCO and PEGASOS whole mouse labeling, the immunostaining buffer and process were adopted from the iDISCO methods. Afterward, the clearing steps of uDISCO and PEGASOS were performed based on the original publications^31,32^.

### Light-sheet microscopy imaging

Image stacks were acquired using a Blaze ultramicroscope (LaVision BioTec GmbH, v.7.3.2) with an axial resolution of 4 μm and the following filter sets: excitation 470/40 nm, emission 535/50 nm; excitation 545/25 nm, emission 605/70 nm and excitation 640/40 nm, emission 690/50 nm. Whole mouse bodies were scanned individually with an UltraMicroscope Blaze light-sheet microscopy ×4 objective (Olympus XLFLUOR ×4 corrected/0.28 numerical aperture (working distance, 10 mm)). We covered the entire mouse in with 9 × 23 tile scans with 20% overlap and imaged them separately from the ventral and dorsal surfaces to a depth of 10 mm, covering the entire body volume with a *z* step of 10 µm. The width of the light sheet was reduced to 60% to achieve maximum illumination of the field of view, and the exposure time was set to 120 ms. The laser power was adjusted as a function of the intensity of the fluorescence signal to avoid saturation. The acquired raw images TIFF were processed with the Fiji stitching plugin (http://www.discotechnologies.org/).

### Reconstructions of full-body scans and quantification

Detailed step-by-step instructions for image data stitching and volume fusion were provided previously^6^. Briefly, image stacks were recorded using ImSpector software (LaVision BioTec GmbH) and saved in TIFF format for each channel separately. The scanned ventral and dorsal-mouse image data were first stitched using the Fiji stitching plugin and volumes fused using Vision4D (v.3.5x64, Arivis AG, v.3.4.0). To increase the precision of volume fusion, alignment was performed by manually selecting three to four anatomical landmarks from the overlapping regions. Representative images were created using Imaris (Bitplane AG, v.9.6.0) and Vision4D for 3D volumetric reconstruction, maximum intensity projection and depth color rendering. To isolate a specific tissue region, the Imaris surface tool was used manually and the mask channel option for pseudocolor was selected. After manual segmentation, the region was visualized in 2D slices using the Ortho Slicer tool. VR pictures and Videos were generated using the Syglass software (IstoVisio, Inc., v.1.7.2).

For quantification of the enteric plexus in the duodenum between germ-free mice and wildtype mice, five 200 × 200 × 200 μm^3^ cubic volumes along the portal triads were randomly selected from the reconstructed 3D images in Imaris. The length of the PGP 9.5-positive enteric plexus in each cubic volume was traced using Imaris Filament Tracer.

### Colocalization analysis

Colocalization analysis was carried out using BitPlane Imaris. To quickly analyze the colocalization ratio, the whole-body data were cropped to specific organ and tissue data. Both filament tracer and surface rendering in Imaris were applied to calculate the colocalization ratios.

### Spatial quantification between TLS and metastases

Given a 3D annotation of TLS and metastases, we used the cc3d library to implement connected component analysis to label every TLS and metastases, and obtain the positions of their centroids. Using the centroid information, we computed the distances to the nearest neighboring metastases, the nearest neighboring TLS and between TLS and metastases. In addition, we calculated the volume and size of every TLS and metastases using the labeled connected component results.

### VR headset procedure

Supplementary Videos 24–31 require a VR headset. To visualize them, you need a VR video player on your VR device or computer. Videos played in VR need to have ‘_360’ at the end of their file name and be set to a ‘360°/3D’ view in the VR player for an immersive experience.

### Whole mouse atlas website construction

We constructed the entire mouse atlas website by stitching together imaging data obtained from light-sheet microscopy using ImageJ/Fiji (v.1.53) and the plugin ‘stich sequence of grids of images’. Afterward, we compressed the entire image dataset using ImageMagick LZW compression software to reduce data size from several terabytes to a few hundred gigabytes. We used the ome-zarr-py tool to convert the compressed TIFF image format to the OME-Zarr image format. The resulting OME-Zarr images are now accessible via webKnossos^40^.

### Quantification

Data are presented as mean ± s.d. Statistical analysis was performed using Prism GraphPad software v.6 with 95% confidence interval. *P* values were calculated using two-tailed unpaired *t*-tests to compare data between two groups. *P* < 0.05 were considered statistically significant.

### Reporting summary

Further information on research design is available in the Nature Portfolio Reporting Summary linked to this article.

## Online content

Any methods, additional references, Nature Portfolio reporting summaries, source data, extended data, supplementary information, acknowledgements, peer review information; details of author contributions and competing interests; and statements of data and code availability are available at 10.1038/s41587-023-01846-0.

## Supplementary information

Supplementary Information Supplementary Figs. 1–11 and Table 1.

Reporting Summary

Supplementary Video 1 3D reconstruction of a mouse labeled with PGP 9.5 and imaged using light-sheet microscopy. Different PGP 9.5 innervation regions (green) are visualized with high contrast over background (gray).

Supplementary Video 2 3D view of the PGP 9.5-positive peripheral nerve in a mouse heart shown in magenta.

Supplementary Video 3 3D visualization of nerves in mouse spleen labeled with PGP 9.5 in magenta.

Supplementary Video 4 3D visualization of mouse liver and gallbladder innervated with PGP 9.5-positive peripheral nerves in magenta.

Supplementary Video 5 3D annotation of neuronal connections in multiple organs (kidney, spleen, liver and intestine) labeled with PGP 9.5 in green.

Supplementary Video 6 3D annotation of mouse intestinal innervation with TH-positive nerve in magenta. Grid-like lattice structures can be seen in the intestinal wall.

Supplementary Video 7 Vagus nerve in magenta tracing in the whole body.

Supplementary Video 8 3D visualization of the entire lymphatic vessels in the whole mouse. The lymphatic vessels of a 4-week-old mouse were labeled with LYVE1 in yellow.

Supplementary Video 9 3D visualization of the hindlimb of a mouse with the LYVE1 lymphatic vessel labeled in yellow. Fine details of the lymphatic vessels and lymph nodes are shown.

Supplementary Video 10 3D visualization of mouse kidney with LYVE1 lymphatic vessels labeled in yellow.

Supplementary Video 11 3D annotation of mouse stomach with LYVE1 lymphatic vessels highlighted in yellow. The fine details of the lymphatic vessels are clearly visible throughout the scan.

Supplementary Video 12 3D annotation of mouse intestine lymphatic vessels with a yellow color labeled by LYVE1 and scanned with light-sheet microscopy. Fine details of the lymphatic vessels can be seen in the intestine and a lymph node is located adjacent to the intestine.

Supplementary Video 13 View of the intestinal wall of a mouse with LYVE1 lymphatic vessels labeled in yellow.

Supplementary Video 14 wildDISCO staining of Prox1 lymphatic vessels in green and arterial staining of alpha-SMA in red, revealing lymphatic vessels penetrated in mouse cerebral cortex.

Supplementary Video 15 3D illustration showing wildDISCO staining of LYVE1 in yellow and podoplanin in magenta to visualize lymphatic capillaries covered on the surface of the mouse brain, and also entering the brain parenchyma around the thalamus.

Supplementary Video 16 3D view of LYVE1-labeled lymphatic vessels entering from the brain to vertebrae.

Supplementary Video 17 Intact mouse with skin labeled with LYVE1 lymphatic vessel.

Supplementary Video 18 3D illustration showing innervation of intestinal lymph nodes by sympathetic neurons using TH staining to label sympathetic nerves in magenta and CD45 staining to label immune cells in green.

Supplementary Video 19 Representative 3D reconstructions of immune cells on the intestine neurons. CD45 is stained in green for immune cell distribution and TH stained in magenta for sympathetic nerves.

Supplementary Video 20 3D reconstruction of a TH and LYVE1-labeled mouse by light-sheet microscopy. Different regions of sympathetic nerve innervation in green and lymphatic vessels in blue can be seen.

Supplementary Video 21 3D illustration of the TH-stained sympathetic nerve in green interacting with LYVE1-labeled lymphatic vessels in yellow on the intestinal wall.

Supplementary Video 22 3D illustration of TH+ sympathetic nerve (green) interacting with LYVE1+ lymphatic vessels (yellow) inside the intestine.

Supplementary Video 23 3D illustration showing the distribution of multiple lymph nodes throughout the mouse. Innervation of pan-neuron marker PGP 9.5 in green and lymph node masked color in cyan.

Supplementary Video 24 Representative 3D illustration of a posterior limb lymph node innervated by PGP 9.5-positive nerves in green and Prox1 positive lymph nodes in magenta.

Supplementary Video 25 TLS stained with CD3 and CD23 on a 4T1 cancer metastasis model.

Supplementary Video 26 Ki67^+^ proliferating cells colocalized with propidium iodide in the spinal cord.

Supplementary Video 27 Ki67^+^ proliferating cells colocalized with propidium iodide in vertebrae.

Supplementary Video 28 Tutorial video for the atlas website.

Supplementary Video 29 Summary of results showing a homogeneous and simultaneous antibody staining throughout the entire mouse bodies.

Supplementary Video 30 VR 3D visualization of neuronal connections in multiple organs labeled with PGP 9.5 in green and masked colors for kidney, spleen, liver and intestine.

Supplementary Video 31 VR 3D visualization of mouse intestinal innervation with TH-positive nerve in magenta. Grid-like structures are clearly visible throughout the intestinal wall.

Supplementary Fig. 1 Statistical source data.

Supplementary Fig. 3 Statistical source data.

Supplementary Fig. 5 Statistical source data.

Supplementary Fig. 6 Statistical source data.

Supplementary Fig. 7 Statistical source data.

Supplementary Fig. 8 Statistical source data.

## Source data

Source Data Fig. 1 Statistical source data.

Source Data Fig. 3 Statistical source data.

Source Data Fig. 4 Statistical source data.

## Data Availability

All data that support the findings of this study are available from the corresponding author upon reasonable request. An atlas of high-resolution images of whole mouse nervous systems is available at http://discotechnologies.org/wildDISCO/atlas/index.php. Source data are provided with this paper.
