# Supplementary material for: Whole-body cellular mapping in mouse using standard IgG antibodies
Source: Nat Biotechnol. 2023 Jul 10;42(4):617–27. doi: 10.1038/s41587-023-01846-0 (PMC11021200; doi:10.1038/s41587-023-01846-0)
Supplement: Supplementary file 1 — Supplementary Figs. 1–11 and Table 1. [file 41587_2023_1846_MOESM1_ESM.pdf]

nature biotechnology

Article

https://doi.org/10.1038/s41587-023-01846-0

Whole-body cellular mapping in mouse using standard IgG antibodies

In the format provided by the authors and unedited

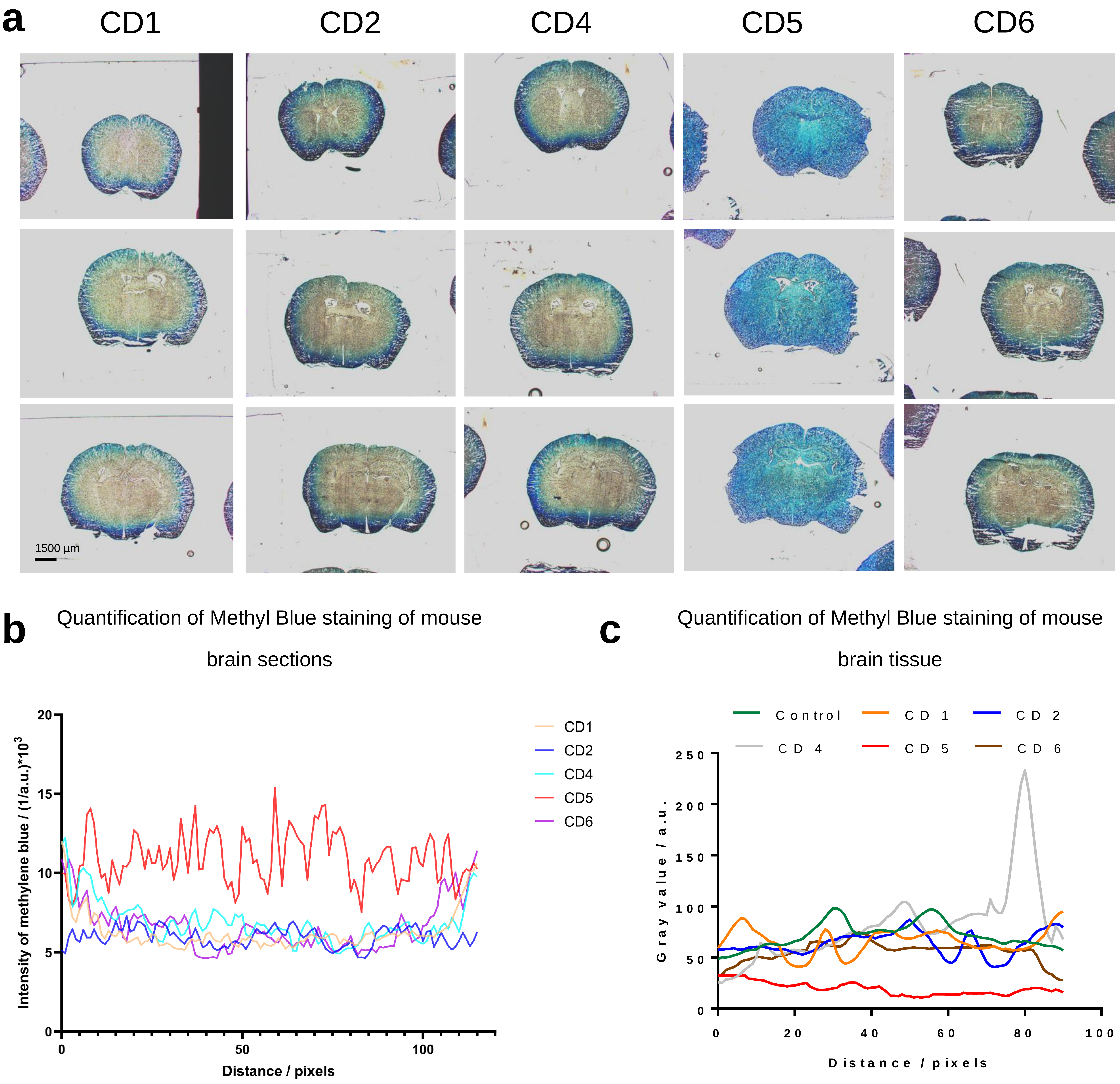

Supplementary Figure 1

Screening the cyclodextrins with Methyl Blue staining to mouse brain section

(a) Methylene blue staining of individual hemispheres of mouse brains after permeabilization with different CD-containing solutions and imaging on different brain frozen sections. CD5 is shown to strongly enhance tissue permeabilization compared to others. n=4. (b) Profile plot along each frozen section of mouse brains in Figure S1a. (c) Profile plot along each mouse brain dimension in Fig. 1c.

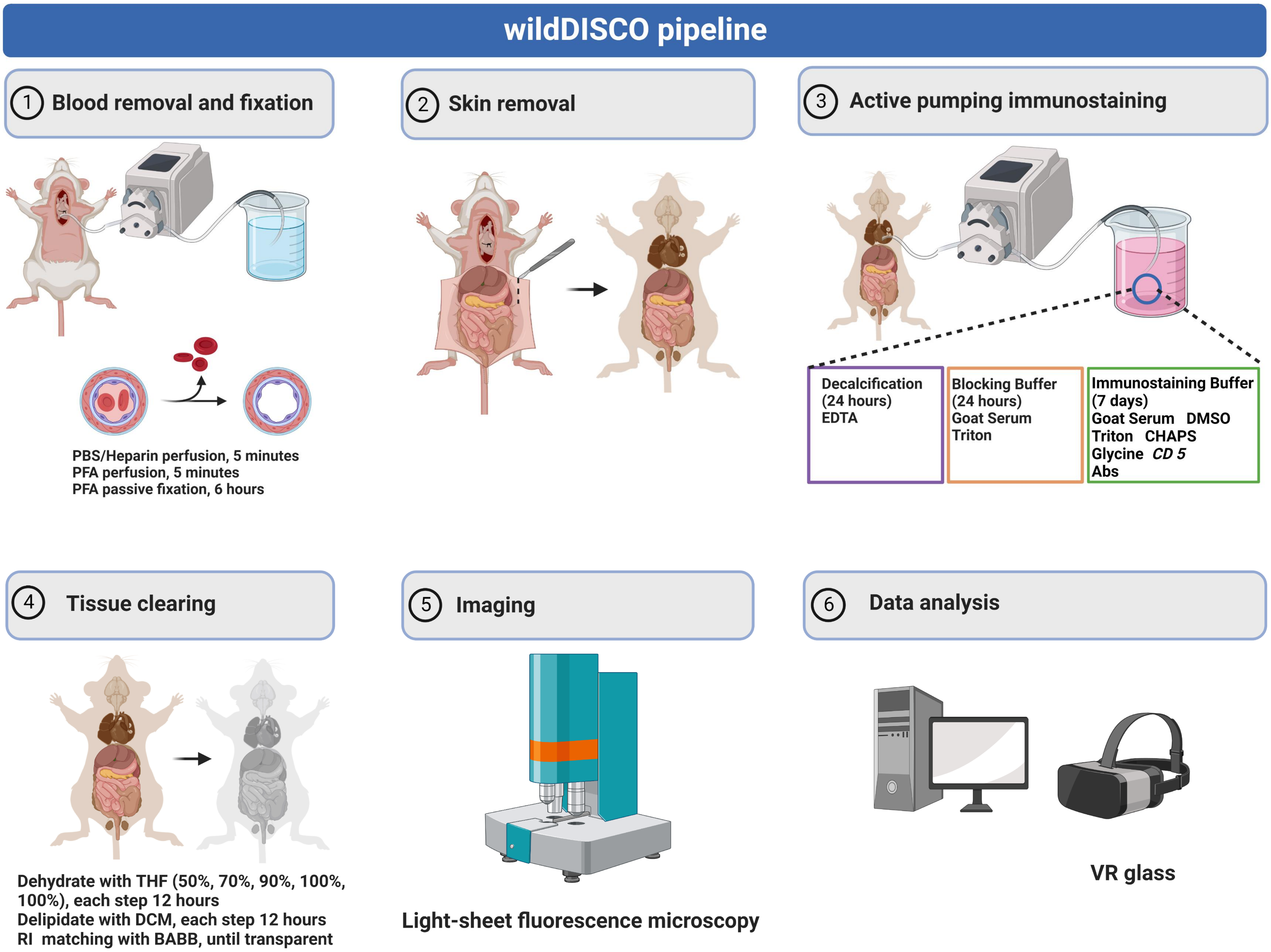

Supplementary Figure 2
Overview of wildDISCO pipeline
Schematic of wildDISCO pipeline.

a

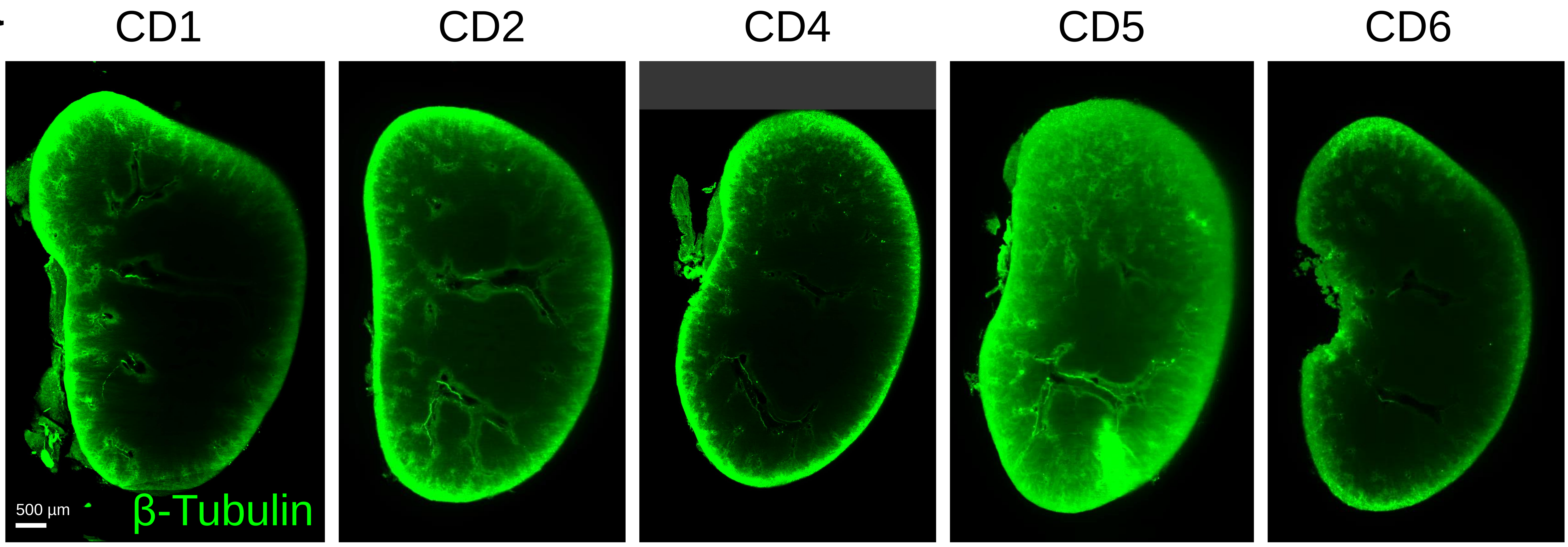

b

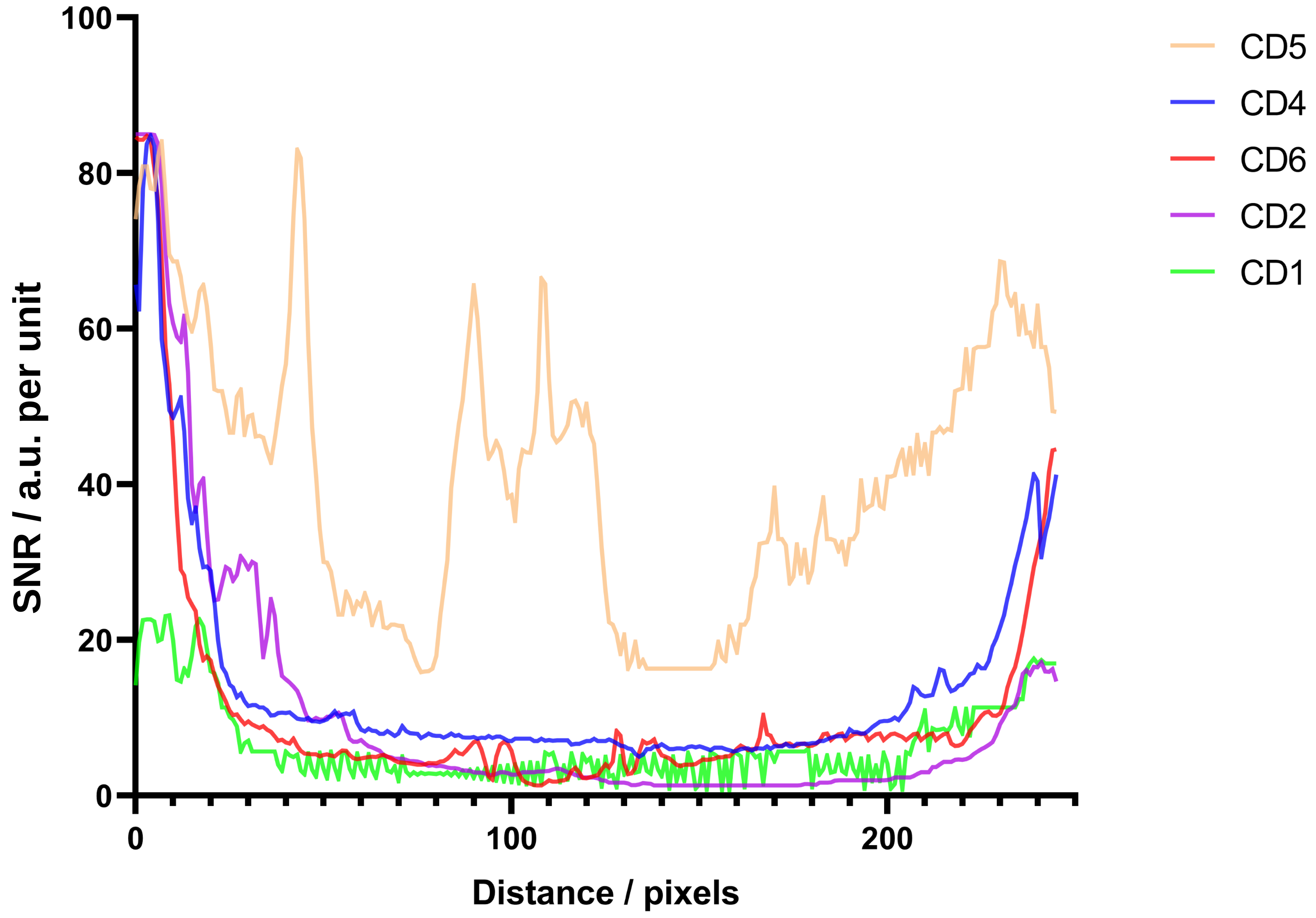

Supplementary Figure 3

Screening CDs for antibody staining of mouse kidney

(a) Representative 2D optical images of beta-tubulin antibody staining of mouse kidney after permeabilization with various CD-containing solutions. CD5 is shown to greatly enhance tissue permeabilization for beta-tubulin antibody compared with others. n=4. (b) Signal to noise ratio of each mouse kidney.

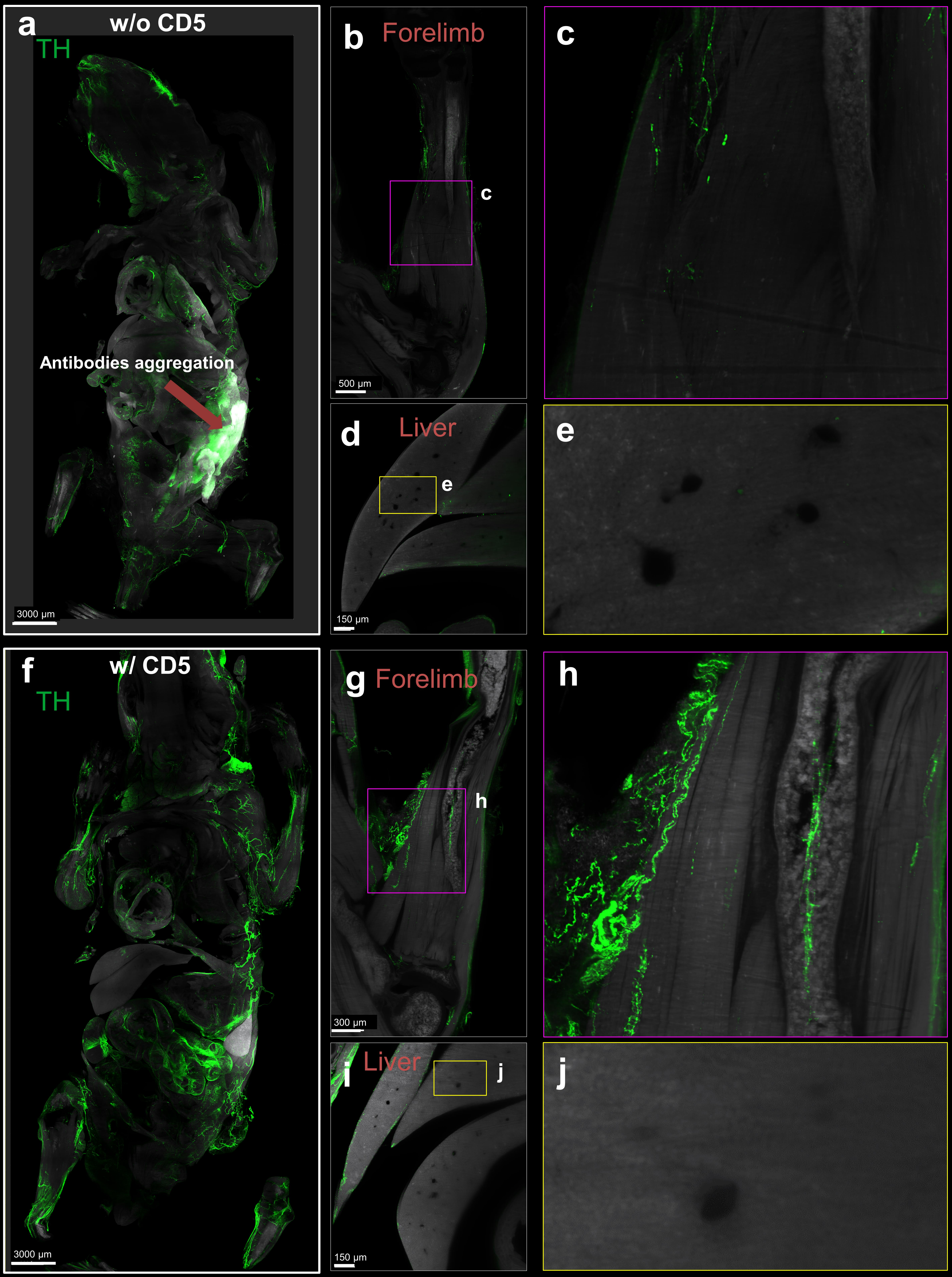

Supplementary Figure 4

Comparison of passive staining with or without CD5 chemical

(a) Passive immunostaining of TH antibody in the whole mouse body without CD5 chemical. Arrow indicated antibody aggregation. (b) Representative images of the passive TH staining in the hindlimb without CD5 chemical treatment. TH antibody is very hard to penetrate in the bone marrow(c). (d-e) There is no TH staining in the liver using passive staining without CD5 treatment. (f) Passive immunostaining of TH antibody in the whole mouse body with CD5 chemical. (g) Representative images of the passive TH staining in the hindlimb with CD5 chemical treatment and showed penetration of TH antibody in the bone marrow(h). (i-j) There is no TH staining in the liver using passive staining even with CD5 treatment. (a-j), n=3.

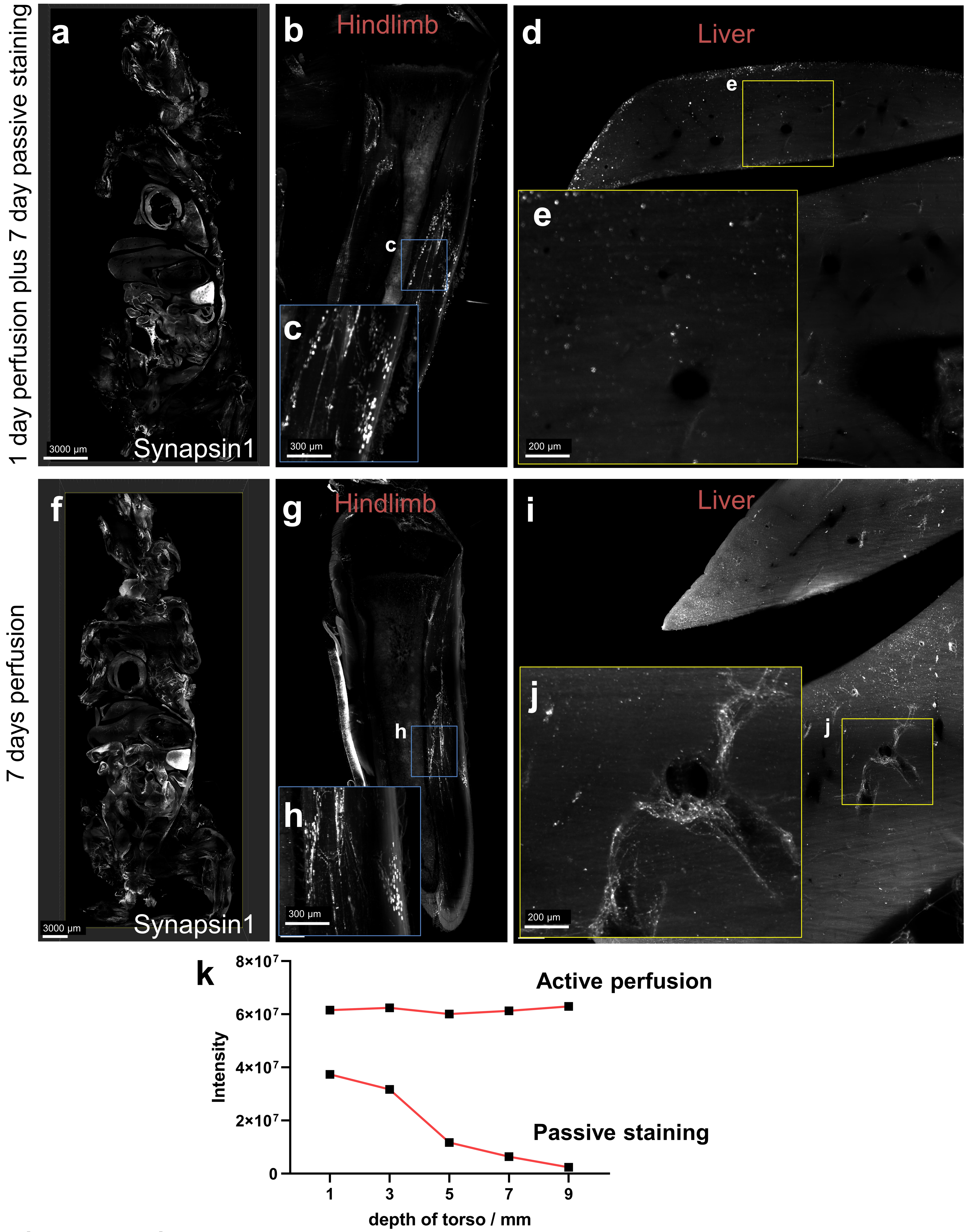

Supplementary Figure 5

Comparison of wildDISCO with active pumping and passive incubation labelling

(a) 2D optical image of immunostaining of the antibody synapsin-1 in the whole mouse body with 1-day perfusion and 7-day passive staining. (b-c) Representative images of hindlimb regions showing synapsin-1-positive neuromuscular junctions (b), with a higher magnification region (c). (d-e) Representative images of the lack of synapsin-1 in the liver with 1 day of perfusion and 7 days of passive staining compared to wildDISCO method 7 days of perfusion. (f) 2D optical image of immunostaining of synapsin-1 antibody in the whole mouse body with wildDISCO 7-day perfusion staining. (g-h) Representative images of synapsin-1 staining in the hindlimb with 7-day perfusion (g), with a higher magnification region (h). (i-j) Representative images of synapsin-1 positive staining in the liver using the wildDISCO method and with a higher magnification region. (a-j), n=3. (k) Profile intensity plot along the depth of each mice liver compared active perfusion and passive staining.

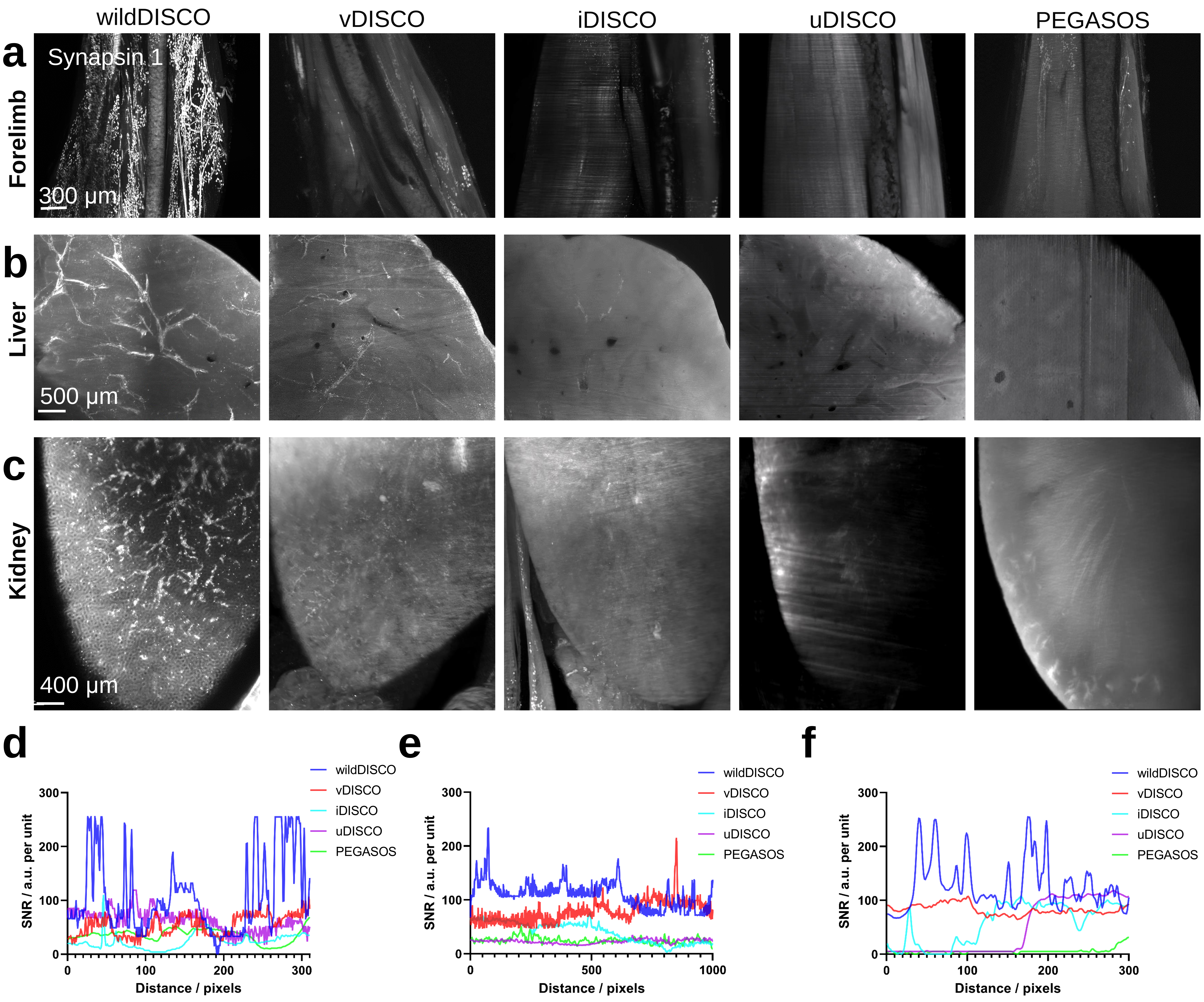

Supplementary Figure 6

Comparison of the different clearing methods using synapsin 1 staining

(a-c) Representative 2D optical images of forelimb (a), liver (b), and kidney (c) stained with synapsin-1 antibodies by wildDISCO, vDISCO, iDISCO, uDISCO, and PEGASOS methods, respectively. n=3. (d-f) Quantification of the depth of penetration of synapsin-1 antibody into the forelimbs (d), liver (e), and kidney (f) of mice using wildDISCO, vDISCO, iDISCO, uDISCO, and PEGASOS, respectively.

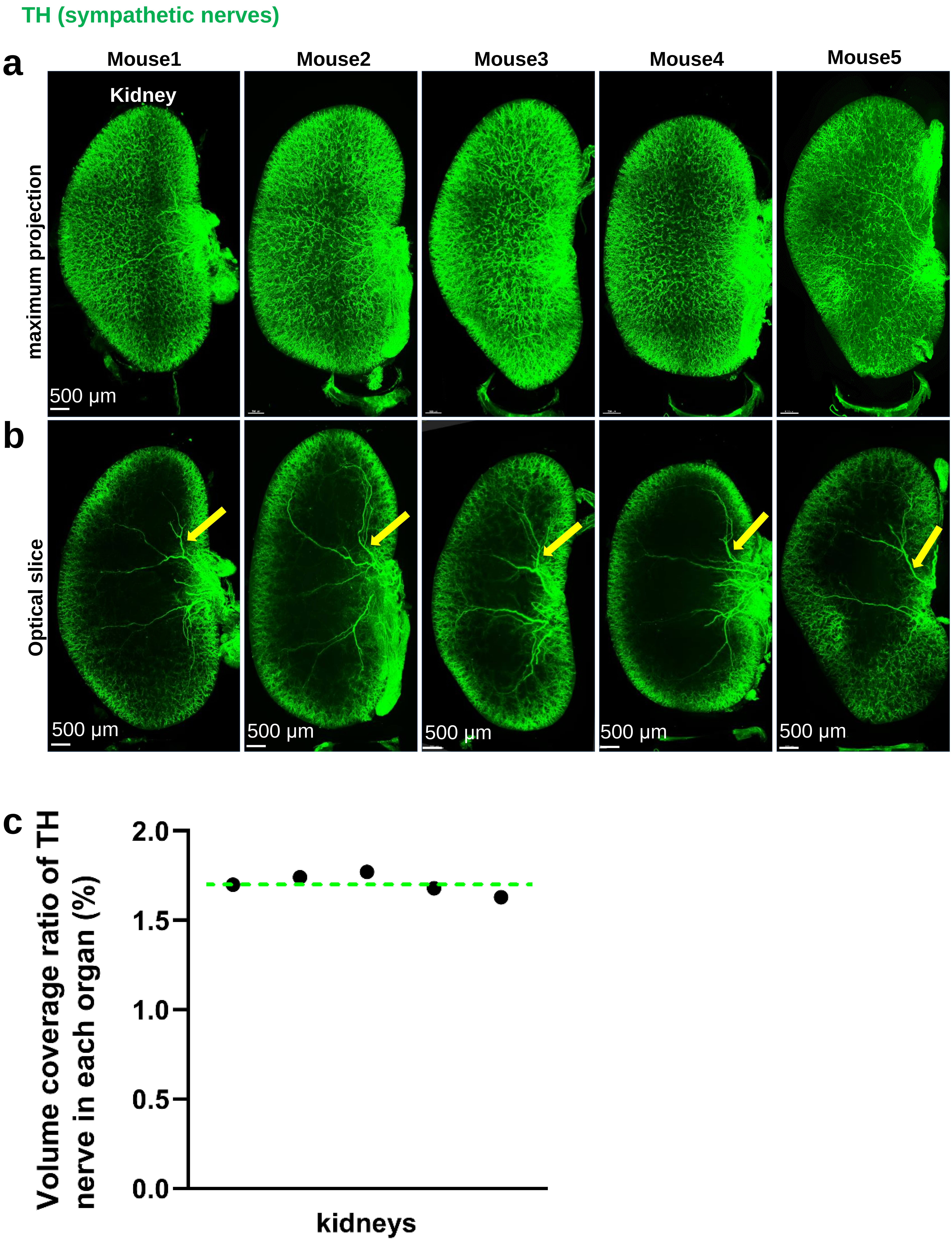

Supplementary Figure 7
Variability of antibody staining in different mice
(a-b) wildDISCO method stained in different individual mice and representative images of maximum projection from different mouse kidneys. n=3. (c) Quantification of the volume coverage of the nerve TH in each mouse kidney and no significant differences.

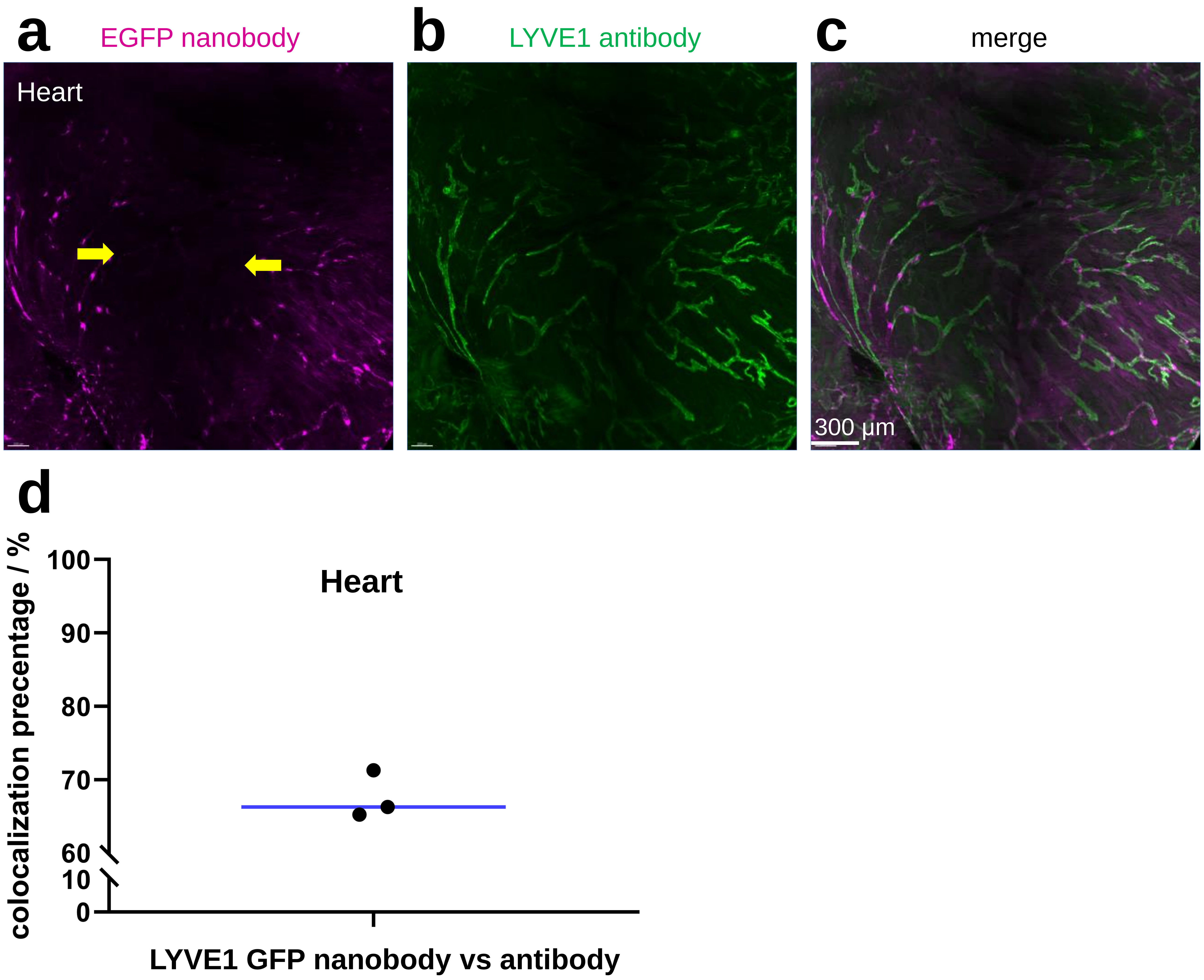

Supplementary Figure 8

Transgenic LYVE1-GFP mouse compare to LYVE1 antibody staining

Immunostaining of Lyve-1 EGFP mice with transgenic fluorescent protein EGFP (a) and Lyve1 protein (b), co-localization (c) in the heart. n=3. (d) Quantification of the percentage of colocalization of Lyve1 EGFP and Lyve1 protein in the heart.

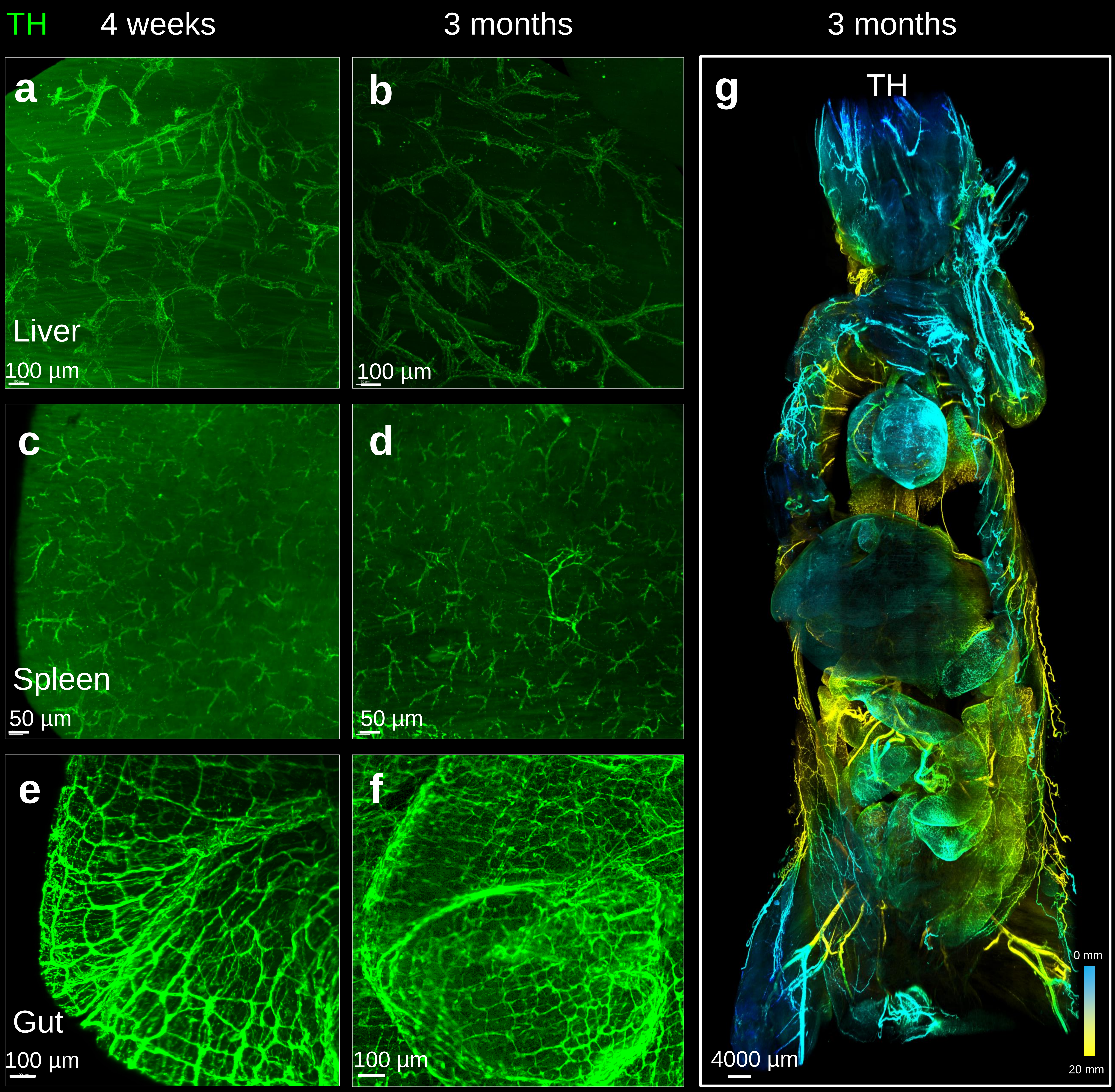

Supplementary Figure 9
wildDISCO is applicable to mice up to 3 months old
(a-b) Representative 3D images of TH-positive staining in the liver using the wildDISCO method on 4 weeks old (a) and 3 months old (b) mouse. (c-d) Representative 3D images of TH-positive staining in the kidney. (e-f) Representative 3D images of TH-positive staining in the gut. (g) wildDISCO-compatible immunostaining of TH in the 3-month-old mouse body (depth color). Experiment was done in triplicate.

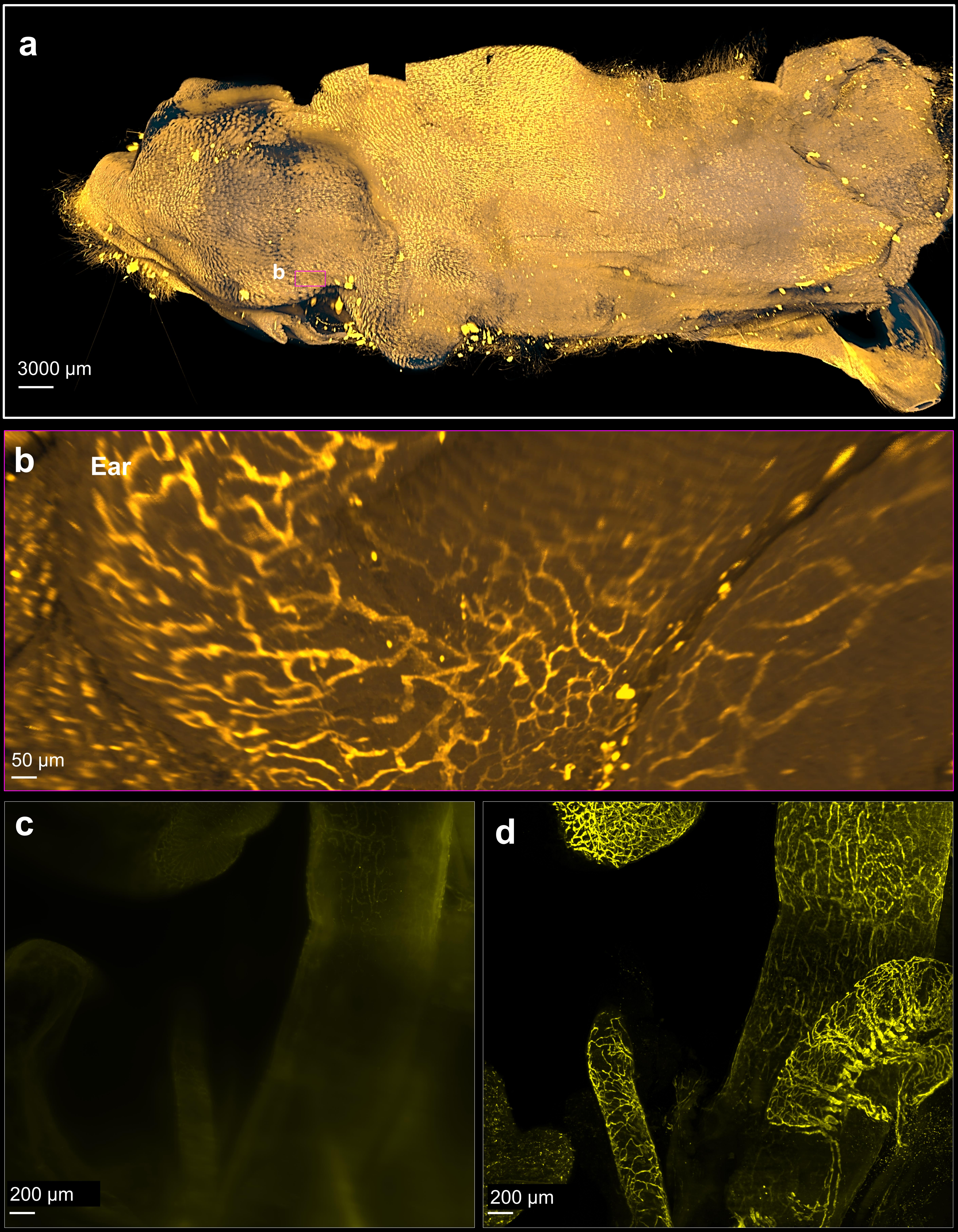

Supplementary Figure 10
Mouse with skin, labeled with LYVE1
(a) A 4-week-old Balb/c mouse was immunolabeled by wildDISCO with LYVE1 for whole mouse lymphatic vessels, and a 3D reconstruction of the whole mouse with skin was created. (b) The lymphatic vessel network in the ear regions is shown. (c) Before skin removal, the penetration of laser power through transparent skin is limited. In the internal organs (such as in the gut), visualization of lymphatic vessel is blurry. (d) After skin removal, the same regions of gut lymphatic vessel can be clearly visualized. n=3.

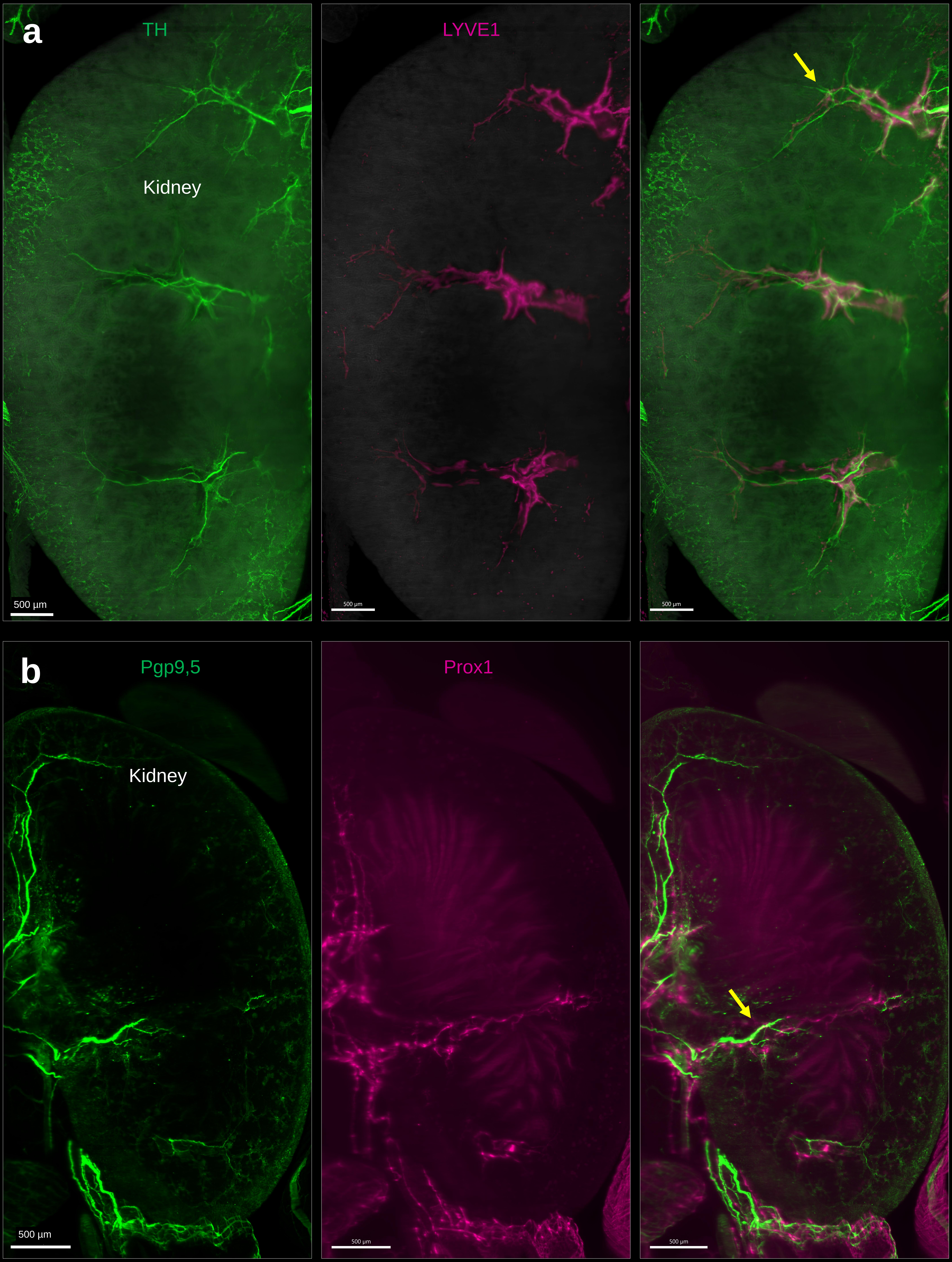

Supplementary Figure 11
Nerve-lymphatic vessel interactions in the kidney
(a) The TH sympathetic neuron (green) innervates the LYVE1 lymphatic vessel (magenta) in the kidney.
(b) The PGP 9.5 pan-neuronal marker (green) is combined with the Prox1 lymphatic vessel marker (magenta). n=3.

Video captions

Supplementary Video 1

3D reconstruction of a mouse labeled with PGP 9.5 and imaged with light-sheet microscopy. Different PGP 9.5 innervation regions (green) are visualized with high contrast over background (gray).

Supplementary Video 2

3D view of PGP 9.5 positive peripheral nerve in mouse heart showing in magenta.

Supplementary Video 3

3D visualization of nerves in mouse spleen labeled with PGP9.5 in magenta.

Supplementary Video 4

3D visualization of mouse liver and gallbladder innervated with PGP 9.5 positive peripheral nerves in magenta.

Supplementary Video 5

3D annotation of neuronal connections in multiple organs (kidney, spleen, liver, and intestine) labeled with PGP 9.5 in green.

Supplementary Video 6

3D annotation of mouse intestinal innervation with TH positive nerve in magenta. Grid-like lattice structures can be seen in the intestinal wall.

Supplementary Video 7

Vagus nerve in magenta tracing at whole body.

Supplementary Video 8

3D visualization of the entire lymphatic vessels in the whole mouse. The lymphatic vessels of a 4-week-old mouse were labeled with LYVE1 in yellow.

Supplementary Video 9

3D visualization of the hindlimb of a mouse with LYVE1 lymphatic vessel labeling in yellow. Fine details of the lymphatic vessels and lymph nodes are shown.

Supplementary Video 10

3D visualization of mouse kidney with LYVE1 lymphatic vessels labeled in yellow.

Supplementary Video 11

3D annotation of mouse stomach with LYVE1 lymphatic vessels highlighted in yellow. The fine details of the lymphatic vessels are clearly visible throughout the scan.

Supplementary Video 12

3D annotation of mouse intestine lymphatic vessels with yellow color labeled by LYVE1 and scanned with light-sheet microscopy. Fine details of the lymphatic vessels can be seen in the intestine and a lymph node is located adjacent to the intestine.

Supplementary Video 13

view of the intestinal wall of a mouse with LYVE1 lymphatic vessels labeled in yellow.

Supplementary Video 14

wildDISCO staining of Prox1 lymphatic vessels in green and arterial staining of alpha-SMA in red, revealing lymphatic vessels penetrated in mouse cerebral cortex.

Supplementary Video 15

3D illustration showing wildDISCO staining of LYVE1 in yellow and podoplanin in magenta to visualize lymphatic capillaries covered on the surface of the mouse brain, and also entering the brain parenchyma around thalamus.

Supplementary Video 16

3D view of LYVE1 labeled lymphatic vessels entering from brain to vertebrae.

Supplementary Video 17

Intact mouse with skin labelled with LYVE1 lymphatic vessel.

Supplementary Video 18

3D illustration showing innervation of intestinal lymph nodes by sympathetic neurons using TH staining to label sympathetic nerves in magenta and CD45 staining to label immune cells in green.

Supplementary Video 19

Representative 3D reconstructions of immune cells on the intestine neurons. CD45 is stained in green for immune cell distribution and TH stained in magenta for sympathetic nerves.

Supplementary Video 20

3D reconstruction of a TH and LYVE1 labeled mouse by light sheet microscopy. Different regions of sympathetic nerve innervation in green and lymphatic vessels in blue can be seen.

Supplementary Video 21

3D illustration of the TH-stained sympathetic nerve in green interacting with LYVE1-labeled lymphatic vessels in yellow on the intestinal wall.

Supplementary Video 22

3D illustration of TH+ sympathetic nerve (green) interacting with LYVE1+ lymphatic vessels (yellow) inside the intestine.

Supplementary Video 23

3D illustration showing the distribution of multiple lymph nodes throughout the mouse. Innervation of pan-neuron markers PGP 9.5 in green and lymph node masked color in cyan.

Supplementary Video 24

Representative 3D illustration of a posterior limb lymph node innervated by PGP 9.5 positive nerves in green and Prox1 positive lymph nodes in magenta.

Supplementary Video 25

TLS stained with CD3 and CD23 on 4T1 cancer metastasis model.

Supplementary Video 26

$Ki67^+$ proliferating cells colocalized with PI in spinal cord.

Supplementary Video 27

$Ki67^+$ proliferating cells colocalized with PI in vertebrae.

Supplementary Video 28

Tutorial video for atlas website.

Supplementary Video 29

Summary of results showing a homogeneous and simultaneous antibody staining throughout the entire mouse bodies.

Supplementary Video 30

VR 3D visualization of neuronal connections in multiple organs labeled with PGP 9.5 in green and masked colors for kidney, spleen, liver, and intestine.

Supplementary Video 31

VR 3D visualization of mouse intestinal innervation with TH positive nerve in magenta. Grid-like structures can be clearly visible throughout the intestinal wall.

Supplementary Table 1

Validated antibodies over 30. In most case we use an antibody concentration of 0.1µg/ml. Each image of antibody staining is listed here http://discotechnologies.org/wildDISCO/

Supplementary Table 1

Validated Antibodies	SOURCE	IDENTIFIER	Price<br>Euro/mouse (20g)
Rabbit Anti-Tyrosine hydroxylase (TH)	Millipore	Cat# AB152	424 euro/100ug<br>106 euro/mouse
Rabbit Anti-Tyrosine hydroxylase (TH)	Abcam	Cat# ab6211	489 euro/100ug<br>122.25 euro/mouse
Rabbit Anti-Tyrosine hydroxylase (TH)	Cell Signaling Technology	Cat# 2792	402 euro/100ug<br>100.5 euro/mouse
Rabbit Anti-PGP 9.5	Proteintech	Cat# 14730-1-AP	293 euro/150ug<br>48.8 euro/mouse
Rabbit Anti-S100 beta	Abcam	Cat# ab52642	531 euro/100ug<br>132.75 euro/mouse
FRabbit Anti-Neurofilament NF-M	Covance	Cat# PRB-575C	185 euro/100ug<br>46.25 euro/mouse
Rabbit Anti-Podoplanin	Life Technologies	Cat#MA529742	324 euro/100ug<br>81 euro/mouse
Rabbit Anti-Peripherin	Merck Millipore	Cat#AB1530	350 euro/100ug<br>87.5 euro/mouse
Rabbit Anti-Synapsin 1	Cell Signaling	Cat#5297S	339 euro/100ug<br>84.75 euro/mouse

Rabbit Anti-Synapsin 2	Cell Signaling	Cat#85852S	332 euro/100ug<br>83 euro/mouse
Alexa Fluor647 anti-Tubulin β 3	BioLegend	Cat#801210	208 euro/100ug<br>52 euro/mouse
Alexa Fluor647 anti-Tubulin β 3	Abcam	Cat#ab190575	527 euro/100ug<br>131.75 euro/mouse
Rabbit Anti-Tubulin	GeneTex	Cat# GTX00877	474 euro/100ug<br>118.5 euro/mouse
Rabbit Anti-β Tubulin	Abcam	Cat# ab179513	486euro/100ug<br>121.5 euro/mouse
Mouse Anti-Podocalyxin	R and D Systems	Cat# MAB1556	304 euro/100ug<br>76 euro/mouse
Rabbit Anti-CD3	Abcam	Cat# ab16669	365 euro/100ug<br>91.25 euro/mouse
Rabbit Anti-CD3	Abcam	Cat# ab5690	538 euro/100ug<br>134.5 euro/mouse
Rat Anti-CD23	eBioscience	Cat# 14-0232-81	50 euro/100ug<br>12.5 euro/mouse
Rabbit Anti-CD23	Abcam	Cat# ab302526	466 euro/100ug<br>116.5 euro/mouse
Rabbit Anti-Ki67	Abcam	Cat# ab15580	547 euro/100ug<br>136.75 euro/mouse

Rabbit Anti-Oct4	Abcam	Cat# ab19857	470 euro/100ug<br>117.5 euro/mouse
Mouse Anti-Nestin	Santa Cruz	Cat# sc-101541	286 euro/200ug<br>35.75 euro/mouse
Rat Anti-Myelin Basic Protein MBP	Sigma-Aldrich	Cat# MAB386	363 euro/1mg<br>9.075 euro/mouse
Rabbit Anti-Sox2	Cell Signaling	Cat#2748	275 euro/100ug<br>68.75 euro/mouse
Rabbit Anti-PAX7	Thermo Fisher Scientific	Cat# PA1-117	455 euro/100ug<br>113.75 euro/mouse
Rabbit Anti-LGR5	Abcam	Cat#ab219107	500 euro/100ug<br>125 euro/mouse
Goat Anti-CGRP	Abcam	Cat#ab36001	501 euro/100ug<br>125.25 euro/mouse
Rabbit Anti-Alpha smooth muscle actin SMA	Abcam	Cat# ab5694	610 euro/100ug<br>152.5 euro/mouse
Rabbit Anti-Collagen IV	Abcam	Cat# ab6586	480 euro/100ug<br>120 euro/mouse
Rabbit Anti-Prox1	Millipore	Cat# AB5475	372 euro/100ug<br>93 euro/mouse
Rabbit Anti-Prox1	Abcam	Cat# ab101851	467 euro/100ug<br>116.75 euro/mouse

Rat Anti-LYVE1	Thermo Fisher Scientific	Cat# 14-0443-82	193 euro/100ug<br>48.25 euro/mouse
Rabbit Anti-Iba1	FUJIFILM Wako shibayagi	Cat# 019-19741	486 euro/50ug<br>243 euro/mouse
Rat Anti-CD45	BD Biosciences	Cat# 14-0451-82	71 euro/100ug<br>17.75 euro/mouse
Rabbit Anti-CD45	Abcam	Cat# ab10558	480 euro/100ug<br>120 euro/mouse
Rabbit Anti-GAP43	Novus Biologicals	Cat# NB300-143	349 euro/100ug<br>87.25 euro/mouse
Rabbit Anti-CD68	Abcam	Cat# ab125212	538 euro/100ug<br>134.5 euro/mouse
Rabbit Anti-PBR peripheral benzodiazepine receptor	Abcam	Cat# ab109497	585 euro/100ug<br>146.25 euro/mouse
Rat Anti-CD4	eBioscience	Cat# 14-0041-82	67 euro/100ug<br>16.75 euro/mouse
Rabbit Anti-CD8	Thermo Fisher Scientific	Cat# PA5114996	430 euro/100ug<br>107.5 euro/mouse
Alexa Fluor 647 Anti-GFP	Thermo Fisher Scientific	Cat# A31852	299 euro/100ug<br>74.75 euro/mouse
Rat Anti-CD19	Thermo Fisher Scientific	Cat# 16-0193-81	69 euro/50ug<br>34.5 euro/mouse

Alexa Fluor Plus 647 goat anti-rabbit IgG antibody	Thermo Fisher Scientific	Cat# A32733	278 euro/1mg<br>6.95 euro/mouse
Alexa Fluor 594 goat anti-rat IgG antibody	Thermo Fisher Scientific	Cat# A-21245	191 euro/500ug<br>9.55 euro/mouse
Alexa Fluor 647 goat anti-rat IgG antibody	Thermo Fisher Scientific	Cat# A-21247	189.9 euro/500ug<br>9.495 euro/mouse
Alexa Fluor 568 goat anti-rabbit IgG antibody	Thermo Fisher Scientific	Cat# A-11036	244 euro/500ug<br>12.2 euro/mouse
Alexa Fluor 568 goat anti-rat IgG antibody	Thermo Fisher Scientific	Cat# A-11077	208 euro/500ug<br>10.4 euro/mouse
