# Supplementary material for: Whole-body cellular mapping in mouse using standard IgG antibodies
Source: Nat Biotechnol. 2023 Jul 10;42(4):617–27. doi: 10.1038/s41587-023-01846-0 (PMC11021200; doi:10.1038/s41587-023-01846-0)
Supplement: Supplementary file 2 — Reporting Summary [file 41587_2023_1846_MOESM2_ESM.pdf]

nature portfolio

Corresponding author(s): Dr. Ali Erturk

Last updated by author(s): May 13, 2023

Reporting Summary

Nature Portfolio wishes to improve the reproducibility of the work that we publish. This form provides structure for consistency and transparency in reporting. For further information on Nature Portfolio policies, see our Editorial Policies and the Editorial Policy Checklist.

Statistics

For all statistical analyses, confirm that the following items are present in the figure legend, table legend, main text, or Methods section.

n/a	Confirmed	
☐	☒	The exact sample size (*n*) for each experimental group/condition, given as a discrete number and unit of measurement
☐	☒	A statement on whether measurements were taken from distinct samples or whether the same sample was measured repeatedly
☐	☒	The statistical test(s) used AND whether they are one- or two-sided<br>*Only common tests should be described solely by name; describe more complex techniques in the Methods section.*
☐	☒	A description of all covariates tested
☐	☒	A description of any assumptions or corrections, such as tests of normality and adjustment for multiple comparisons
☐	☒	A full description of the statistical parameters including central tendency (e.g. means) or other basic estimates (e.g. regression coefficient) AND variation (e.g. standard deviation) or associated estimates of uncertainty (e.g. confidence intervals)
☐	☒	For null hypothesis testing, the test statistic (e.g. *F*, *t*, *r*) with confidence intervals, effect sizes, degrees of freedom and *P* value noted<br>*Give P values as exact values whenever suitable.*
☒	☐	For Bayesian analysis, information on the choice of priors and Markov chain Monte Carlo settings
☒	☐	For hierarchical and complex designs, identification of the appropriate level for tests and full reporting of outcomes
☒	☐	Estimates of effect sizes (e.g. Cohen's *d*, Pearson's *r*), indicating how they were calculated

Our web collection on statistics for biologists contains articles on many of the points above.

Software and code

Policy information about availability of computer code

Data collection	Data collection was performed using: ImSpector (Version 7.3.2, MiltenyiBioTec GmbH), Vision4D (Version 3.4.0, Arivis AG), Imaris (Version 9.6.0 Bitplane AG), syGlass(version 1.7.2), Living Image Software (Version 4.2 Caliper Life Sciences), ImageMagick LZW compression software (Version 7.0.5 ImageMagick Studio LLC)
Data analysis	Data analysis was performed using: Fiji (version 1.53) , GraphpadPrism (version 6).

For manuscripts utilizing custom algorithms or software that are central to the research but not yet described in published literature, software must be made available to editors and reviewers. We strongly encourage code deposition in a community repository (e.g. GitHub). See the Nature Portfolio guidelines for submitting code & software for further information.

Data

Policy information about availability of data

All manuscripts must include a data availability statement. This statement should provide the following information, where applicable:

- Accession codes, unique identifiers, or web links for publicly available datasets
- A description of any restrictions on data availability
- For clinical datasets or third party data, please ensure that the statement adheres to our policy

Data availability
All data that support the findings of this study are available from the corresponding author upon reasonable request. Source data are provided with this paper.
An atlas of high-resolution images of whole mouse nervous, lymphatic, and vascular systems is available at http://discotechnologies.org/wildDISCO/atlas/index.php
Code availability

The code used to analyze the data is available from the corresponding author upon reasonable request.

Field-specific reporting

Please select the one below that is the best fit for your research. If you are not sure, read the appropriate sections before making your selection.

☒ Life sciences ☐ Behavioural & social sciences ☐ Ecological, evolutionary & environmental sciences

For a reference copy of the document with all sections, see nature.com/documents/nr-reporting-summary-flat.pdf

Life sciences study design

All studies must disclose on these points even when the disclosure is negative.

Sample size	ROIs from different parts of the tissue section were used for quantification of volume. Number of ROIs were noted in the figure legends.
Data exclusions	No collected data were excluded.
Replication	At least three replicates were performed with independent measurement and analysis.
Randomization	Randomization was not necessary for this study.
Blinding	Blinding was not pertinent to this study as experimental conditions were mostly evident from the image data.

Reporting for specific materials, systems and methods

We require information from authors about some types of materials, experimental systems and methods used in many studies. Here, indicate whether each material, system or method listed is relevant to your study. If you are not sure if a list item applies to your research, read the appropriate section before selecting a response.

Materials & experimental systems

n/a	Involved in the study	
☐	☒	Antibodies
☐	☒	Eukaryotic cell lines
☒	☐	Palaeontology and archaeology
☐	☒	Animals and other organisms
☒	☐	Human research participants
☒	☐	Clinical data
☒	☐	Dual use research of concern

Methods

n/a	Involved in the study	
☒	☐	ChIP-seq
☒	☐	Flow cytometry
☒	☐	MRI-based neuroimaging

Antibodies

Antibodies used	Anti-Tyrosine hydroxylase (TH) (Millipore, Cat# AB152) Anti-Tyrosine hydroxylase (TH) (Abcam, Cat# ab6211) Anti-Tyrosine hydroxylase (TH) (Cell Signaling Technology, Cat# 2792) Anti-PGP 9.5 (Proteintech, Cat# 14730-1-AP) Anti-S100 beta (Abcam, Cat# ab52642) Anti-Neurofilament NF-M (Covance, Cat# PRB-575C) Anti-Podoplanin (Life Technologies, Cat#MA529742) Anti-Peripherin (Merck Millipore, Cat#AB1530) Anti-Synapsin 1 (Cell Signaling, Cat#5297S) Anti-Synapsin 2 (Cell Signaling, Cat#85852S) Alexa Fluor647 anti-Tubulin β3 (BioLegend, Cat#801210) Alexa Fluor647 anti-Tubulin β3 (Abcam, Cat#ab190575) Anti-Tubulin (GeneTex Cat# GTX00877) Anti-β Tubulin (Abcam Cat# ab179513) Anti-Podocalyxin (R and D Systems, Cat# MAB1556) Anti-CD3 (Abcam Cat# ab16669) Anti-CD3 (Abcam, Cat# ab5690) Anti-CD23 (eBioscience, Cat# 14-0232-81) Anti-CD23 (Abcam, Cat# ab302526) Anti-Ki67 (Abcam, Cat# ab15580) Anti-Oct4 (Abcam, Cat# ab19857) Anti-Nestin (Santa Cruz, Cat# sc-101541) Anti-Myelin Basic Protein MBP (Sigma-Aldrich, Cat# MAB386) Anti-Sox2 (Cell Signaling, Cat#2748) Anti-PAX7 (Thermo Fisher Scientific, Cat# PA1-117) Anti-LGR5 (Abcam, Cat#ab219107) Anti-CGRP (Abcam, Cat#ab36001) Anti-Alpha smooth muscle actin SMA (Abcam, Cat# ab5694) Anti-Collagen IV (Abcam, Cat# ab6586) Anti-Prox1 (Millipore, Cat# AB5475) Anti-Prox1 (Abcam, Cat# ab101851) Anti-LYVE1 (Thermo Fisher Scientific, Cat# 14-0443-82) Anti-Iba1 (FUJIFILM Wako shibayagi, Cat# 019-19741) Anti-CD45 (BD Biosciences, Cat# 14-0451-82) Anti-CD45 (Abcam, Cat# ab10558) Anti-GAP43 (Novus Biologicals, Cat# NB300-143) Anti-CD68 (Abcam, Cat# ab125212) Anti-PBR peripheral benzodiazepine receptor (Abcam, Cat# ab109497) Anti-CD4 (eBioscience, Cat# 14-0041-82) Anti-CD8 (Thermo Fisher Scientific, Cat# PA5114996) Alexa Fluor 647 Anti-GFP (Thermo Fisher Scientific, Cat# A31852) Anti-CD19 (Thermo Fisher Scientific, Cat# 16-0193-81) Alexa Fluor Plus 647 goat anti-rabbit IgG antibody (Thermo Fisher Scientific, Cat# A32733) Alexa Fluor 594 goat anti-rat IgG antibody (Thermo Fisher Scientific, Cat# A-21245) Alexa Fluor 647 goat anti-rat IgG antibody (Thermo Fisher Scientific, Cat# A-21247) Alexa Fluor 568 goat anti-rabbit IgG antibody (Thermo Fisher Scientific, Cat# A-11036) Alexa Fluor 568 goat anti-rat IgG antibody (Thermo Fisher Scientific, Cat# A-11077) All the antibodies were used in 25 µg for a whole mouse staining.
Validation	Anti-Tyrosine hydroxylase (TH) (RRID:AB_390204) Anti-Tyrosine hydroxylase (TH) (RRID:AB_2240393) Anti-Tyrosine hydroxylase (TH) (RRID:AB_2303165) Anti-PGP 9.5 (RRID:AB_2210497) Anti-S100 beta (RRID:AB_882426) Anti-Neurofilament NF-M (RRID:AB_291700) Anti-Podoplanin (RRID:AB_2785565) Anti-Peripherin (RRID:AB_90725) Anti-Synapsin 1 (RRID:AB_2616578) Anti-Synapsin 2

(RRID:AB_2800065) Alexa Fluor647 anti-Tubulin β3 (RRID:AB_2686931) Anti-Podocalyxin (RRID:AB_2166010) Anti-CD3 (RRID:AB_443425) Anti-CD3 (RRID:AB_305055) Anti-CD23 (RRID:AB_467160) Anti-Ki67 (RRID:AB_443209) Anti-Oct4 (RRID:AB_445175) Anti-Nestin (RRID:AB_1126570) Anti-Myelin Basic Protein MBP (RRID:AB_94975) Anti-Sox2 (RRID:AB_823640) Anti-PAX7 (RRID:AB_2539886) Anti-CGRP (RRID:AB_725807) Anti-Alpha smooth muscle actin SMA (RRID:AB_2223021) Anti-Collagen IV (RRID:AB_305584) Anti-Prox1 (RRID:AB_177485) Anti-Prox1 (RRID:AB_10712211) Anti-LYVE1 (RRID:AB_1633414) Anti-Iba1 (RRID:AB_839504) Anti-CD45 (RRID:AB_467251) Anti-CD45 (RRID:AB_442810) Anti-GAP43 (RRID:AB_10001196) Anti-CD68 (RRID:AB_10975465) Anti-PBR peripheral benzodiazepine receptor (RRID:AB_10862345) Anti-CD4 (RRID:AB_467063) Anti-CD8 (RRID:AB_2899632) Alexa Fluor 647 Anti-GFP (RRID:AB_162553) Anti-CD19 (RRID:AB_657669) Alexa Fluor Plus 647 goat anti-rabbit IgG antibody (RRID:AB_2633282) Alexa Fluor 594 goat anti-rat IgG antibody (RRID:AB_2535813) Alexa Fluor 647 goat anti-rat IgG antibody (RRID:AB_141778) Alexa Fluor 568 goat anti-rabbit IgG antibody (RRID:AB_10563566) Alexa Fluor 568 goat anti-rat IgG antibody (RRID:AB_2534121)
All of the antibodies used in this study were obtained from commercial sources, as described, and were validated by the respective manufacturer. Detailed information about the validation and references for each antibody can be accessed through the vendor websites, which can be reached via the catalog numbers listed above. Based on the vendor websites or several references, most of the antibodies used in this study have been tested and found to be suitable for IHC/IF applications. In our experiments, the imaging patterns of the antibodies were found to be consistent with the reference IF patterns and matched the expected distribution based on the existing literature, for both direct and indirect immunolabeling. These findings provide additional support for the validity and accuracy of the antibody labeling in our study.

Eukaryotic cell lines

Policy information about cell lines

Cell line source(s)	4T1 Cell line was purchased from ATCC (ATCC CRL-2539™).
Authentication	ATCC authenticated the cells.
Mycoplasma contamination	Cell lines were not tested for mycoplasma contamination.
Commonly misidentified lines (See ICLAC register)	No commonly misidentified cell lines were used.

Animals and other organisms

Policy information about studies involving animals; ARRIVE guidelines recommended for reporting animal research

Laboratory animals	4-week-old and 3-month-old mixed-gender wildtype mice (C57BL/6J, CD1 and Balb/c) were purchased from Charles River Laboratories, C57BL/6J germ-free mice were purchased from the Technical University of Munich, Institute of Nutrition and Health, Core Facility Gnotobiology
Wild animals	This study does not involve wild animals.
Field-collected samples	This study does not involve field-collected samples.
Ethics oversight	Animal experiments were performed according to the institutional guidelines of the Ludwig Maximilian University of Munich and the Helmholtz Munich Center German Mouse Clinic after approval of the Ethical Review Board of the Government of Upper Bavaria (Regierung von Oberbayern, Munich, Germany).

Note that full information on the approval of the study protocol must also be provided in the manuscript.
